# Antioxidant and anti-inflammatory activity of constituents isolated from *Dendrobium nobile* (Lindl.)

**DOI:** 10.3389/fchem.2022.988459

**Published:** 2022-10-04

**Authors:** Hui Lei, Shunmei Zou, Jiafu Lin, Longfei Zhai, Yifeng Zhang, Xiujuan Fu, Siwei Chen, Hong Niu, Feifei Liu, Chunlian Wu, Dan Zhang

**Affiliations:** ^1^ School of Pharmacy, Southwest Medical University, Luzhou, Sichuan, China; ^2^ School of Pharmacy, Chengdu University, Chengdu, Sichuan, China; ^3^ Antibiotics Research and Re-evaluation Key Laboratory of Sichuan Province, Sichuan Industrial Institute of Antibiotics, Chengdu University, Chengdu, Sichuan, China; ^4^ School of Life Sciences, Jiangsu Normal University, Xuzhou, Jiangsu, China; ^5^ Key Laboratory of Southwest China Wildlife Resources Conservation (China West Normal University), Ministry of Education, Nanchong, Sichuan, China

**Keywords:** *Dendrobium nobile* Lindl., chemical constituents, antioxidant, anti-inflammatory activities, structure-activity relationship

## Abstract

*Dendrobium nobile* (Lindl.) have long been used as herbal tea and a traditional herbal medicine to treat Alzheimer’s disease (AD). In the current study, nineteen compounds (**1–19**), including two new vitamin E homologues (**1–2**), one new sesquiterpene (**6**), and two new dendrobines (**7**, **8**), were isolated and identified from stems of *Dendrobium nobile*. Their structures were elucidated on the basis of NMR, ^13^C NMR calculation, and DP4^+^ probability analyses. The absolute configurations of new compounds were determined by electronic circular dichroism (ECD) data analysis. Antioxidant, anti-inflammatory, and cytotoxic activities of isolated compounds were evaluated. Among them, compound **2** demonstrated significant antioxidant activity compared with ascorbic acid (VC), while compounds **2** and **4** also exhibited an equal effect to positive control cisplatin. This study on the biological activity of the new vitamin E homologues from *Dendrobium nobile* may indicate its potential application in the pharmaceutical and food industries.

## Introduction

**FIGURE 1:**
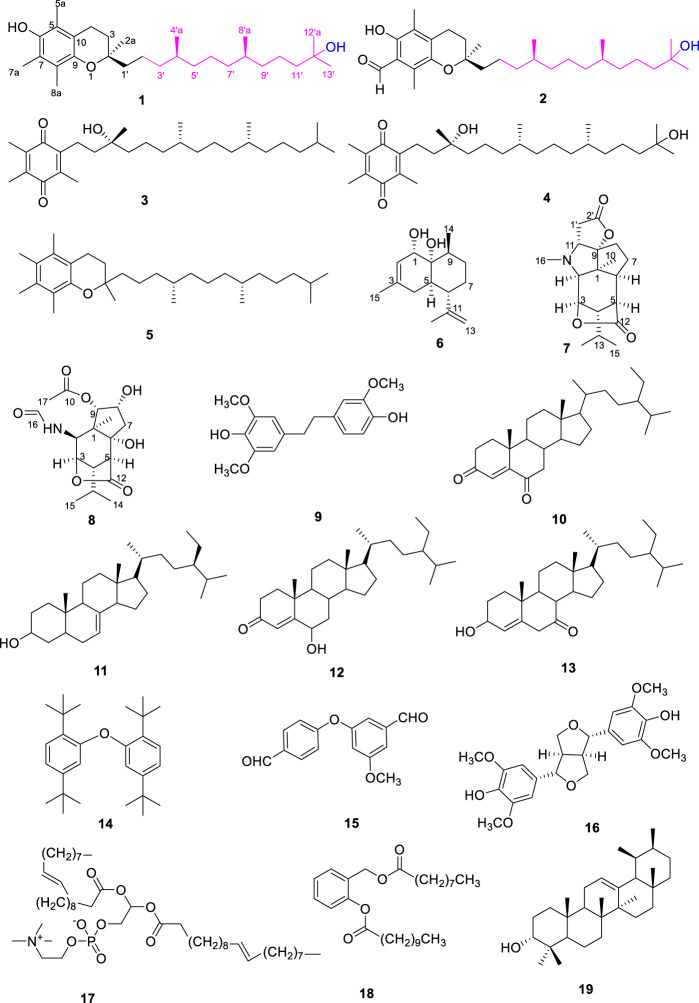
Structures of compounds **1**–**19**.

*Dendrobium* plants (Orchidaceae) are mainly distributed throughout Asia and the Pacific islands. There are 81 species of *Dendrobium* in China, mostly distributed in the South of the Tsinling Mountains of China (Zheng et al., 2018; Wang, 2021a). Owing to their well-known nutritional value and medicinal properties, the genus *Dendrobium* (Orchidaceae) have attracted interest in many health products and pharmaceutical fields (Teixeira da Silva and Ng, 2017). The species *Dendrobium nobile* Lindl, an edible, ornamental, and also a medicinal plant, is one of the four well-known plant sources of “Shi Hu”, mainly distributed in southwest China, as Sichuan, Guizhou, and Yunnan (Wang, 2021b). Abundant bioactive constituents of this plant have been previously obtained, including sesquiterpenes (Ling et al., 2021), phenanthrene, bibenzyl derivatives (Cheng et al., 2020), glucosides (Zhao et al., 2001), and alkaloids (Liu and Zhao, 2003). In order to further reveal and utilize the potential value of *Dendrobium nobile* as an industrial crop used in the pharmaceutical and food industries, we conducted the current study. As a result, nineteen compounds (**1–19**) (Figure 1), including two new vitamin E homologues (**1–2**), one new sesquiterpene (**6**), and two new dendrobines (**7**, **8**), were isolated and identified from the stems of *D. nobile*. In addition, the antioxidant, anti-inflammatory, and anti-tumour activities of the compounds (**1–19**) were evaluated. These outstanding properties of *Dendrobium nobile* will better expand its applications in antioxidant, anti-inflammatory, and anti-tumour activities area, especially in the field of pharmaceutics and food industries.

## Materials and methods

### General experimental procedures

Optical rotations were measured on an AntonPaar MCP500 polarimeter. IR spectra were performed on a Shimadzu IR spectrometer with KBr disk. HRESIMS were used for a Bruker maXis TOF-Q mass spectrometer. Bruker DRX-500 spectrometer was used to measure the NMR spectra. The UV spectra were recorded on a Shimadzu UV-2600 PC spectrometer. Silica gel (100–200 mesh, 200–300 mesh, Qingdao, China), YMC*GEL ODS-A (S-50 μm, 12 nm) (YMC Co., Ltd., Kyoto, Japan), Sephadex LH-20 (GE, Sweden) were used for column chromatography. Semipreparative HPLC was used for ODS column (YMC-ODS-A). CD spectra were performed on a Biologic MOS-450 spectra polarimeter.

### Plant material

The stems of *Dendrobium nobile* were provided by the Chengming Shihu Industrial Development in October, 2020. The sample was botanically identified by Prof. Dan Zhang. A voucher specimen (No. GFM20201024) has been deposited at the Processing and Preparation Laboratory of Traditional Chinese Medicine, Southwest Medical University (Luzhou, China).

### Extraction and isolation

The air-dried stems (2 kg) of *Dendrobium nobile* were crushed and extracted with 95% EtOH (4 × 20 L, 3 days each). After the solvent was evaporated under reduced pressure. The extract (172.6 g) was subjected to column chromatography (CC) over MCI resin and eluted with EtOH-H_2_O (50, 70, 90 and 100%, v/v), to obtain four fractions (Fr.1- Fr.4).

Fr. 1 (62.6 g) was subjected to silica gel column chromatography with gradient elution using methylene chloride/methanol (40:1 to 0:1, v/v) to give eleven subfractions (Fr. 1-1 and Fr.1–11). Fr. 1–2 (1.9 g) was separated by silica gel column with petroleum ether/ethyl acetate (from 15:1 to 0:1, v/v) to yield Fr.1-2-1∼Fr.1-2-10. Fr.1-2-2 was analyzed by TLC, and the preparation condition was dichloromethane: acetone = 40:1 to obtain compound **16** (6.9 mg). Compound **17** (tR = 25 min, 47.3 mg) and compound **18** (tR = 32 min, 13.7 mg) were purified from Fr.1-2-3, using HPLC (MeOH/H_2_O, 55:45, v/v). Fr.1-2-4 was isolated by HPLC (MeOH/H_2_O, 55:45, v/v) to obtain compound **19** (tR = 45 min, 23.7 mg).

Fr. 2 (6.2 g) was isolated into five subfractions (Fr. 2-1 and Fr.2–5) through silica gel column chromatography with gradient elution using petroleum ether/ethyl acetate (30:1 to 0:1, v/v). Compound **15** (5.9 mg) was purified from Fr. 2-3, and the preparation condition was dichloromethane: acetone (40:1).

Fr. 3 (29.7 g) was separated by silica gel column chromatography with gradient elution using petroleum ether/ethyl acetate (30:1 to 0:1, v/v) to produce nine subfractions (Fr. 3-1 and Fr.3–9). Fr. 3-1 was analyzed by TLC, and further purified by preparative TLC with dichloromethane/acetone (40:1) to afford compounds **3** (6.2 mg) and **4** (7.1 mg). Fr. 3–2 (599 mg) was fractionated into four subfractions (Fr. 3-2-1 and Fr.3-2–4) by silica gel column chromatography with gradient elution using petroleum ether/ethyl acetate (15:1 to 0:1, v/v). Fr. 3-2-4 was separated by Sephadex LH-20 column and purified by PTLC with petroleum ether/ethyl acetate (3:1) to obtain compounds **5** (9.3 mg) and **6** (5.9 mg). Fr. 3–5 (1.7035 g) was eluted with silica gel column chromatography using petroleum ether/ethyl acetate (10:1 to 0:1, v/v) and was further separated by PTLC and Sephadex LH-20 (MeOH) to obtain compounds **8** (15.2 mg), **9** (6.4 mg), and **10** (3.6 mg). Fr. 3-5-4 was successively subjected to silica gel CC (dichloromethane/acetone, 40:1) and PTLC to get compounds **11** (5.6 mg) and **7** (18.9 mg). Fr.3-7 was prepared by both silica gel column chromatography and PTLC to obtain compound **12** (9.3 mg). Fr.3-8 was prepared by silica gel column chromatography and PTLC to obtain compound **13** (12.8 mg). Fr.3-9 was prepared by silica gel column chromatography and PTLC to obtain compound **14** (5.4 mg).

Fr. 4 (16.3 g) was chromatographed using silica gel CC and eluted by petroleum ether/ethyl acetate (30:1 to 0:1, v/v) to provide six subfractions (Fr. 4-1 and Fr.4–6). Fr. 4-3 was further fractionated through Sephadex LH-20 (MeOH) as well as PTLC (dichloromethane/acetone, 40:1) to obtain compounds **1** (5.1 mg) and **2** (4.0 mg).

#### 12′-hydroxy-α-tocopherol (**1**)

Yellow oil; [α]^25^_D_-2.1 (c 0.1, MeOH); UV (MeOH) λmax (log *ε*) 290 (2.64) nm; IR (film)νmax 3430, 2970, 1760, 1450, 1360, 1218, 1190, 1118, 1080, 990, cm^−1^; ^1^H NMR and ^13^C NMR data, see Table 1; HRESIMS at *m/z* 445.3638 [M-H]^-^ (calcd for C_29_H_49_O_3_, 445.3690).

**TABLE 1:** ^1^H and^13^C NMR (600 MHz) data for **1** and **2** in CDCl_3_.

No.	1		2	
	δ_C_, type	δ_H_ (J in Hz)	δ_C_, type	δ_H_ (J in Hz)
1	—	—	—	—
2	74.5, C	—	75.2, C	—
3	31.6, CH_2_	1.78, m	30.8, CH_2_	1.82, m
4	20.8, CH_2_	2.60, t (6.9)	18.4, CH_2_	3.03, t (6.8)
5	118.6, C	—	124.2, C	—
6	144.5, C	—	155.8, C	—
7	121.1, C	—	114.5, C	—
8	122.6, C	—	138.4, C	—
9	145.5, C	—	144.0, C	—
10	117.4, C	—	117.6, C	—
1′	39.7, CH_2_	1.53, m	39.5, CH_2_	1.55, m
2′	21.0, CH_2_	1.39, m	20.9, CH_2_	1.40, m
3′	37.6, CH_2_	1.27, m	37.6, CH_2_	1.26, m
4′	32.8, CH	1.39, m	32.8, CH	1.39, m
5′	37.5, CH_2_	1.27, m	37.4, CH_2_	1.26, m
6′	24.5, CH_2_	1.06, m	24.5, CH_2_	1.06, m
7′	37.4, CH_2_	1.07, m	37.4, CH_2_	1.08, m
8′	32.7, CH	1.39, m	32.7, CH	1.39, m
9′	37.4, CH_2_	1.07, m	37.4, CH_2_	1.08, m
10′	21.8, CH_2_	1.30, m	21.8, CH_2_	1.30, m
11′	44.3, CH_2_	1.43, m	44.3, CH_2_	1.43, m
12′	71.2, C	—	71.1, C	—
13′	29.3, CH_3_	1.25, m	29.7, CH_3_	1.25, m
Me-2a	23.8, CH_3_	1.23, m	23.7, CH_3_	1.23, s
Me-5a	11.3, CH_3_	2.11, s	11.3, CH_3_	2.15, s
Me-7a	12.3, CH_3_	2.16, s	194.0, C	10.20, s
Me-8a	11.8, CH_3_	2.14, s	13.2, CH_3_	2.17, s
Me-4′a	19.7, CH_3_	0.85, d (6.6)	19.7, CH_3_	0.85, d (6.6)
Me-8′a	19.7, CH_3_	0.86, d (6.6)	19.7, CH_3_	0.86, d (6.3)
Me-12′a	29.2, CH_3_	1.18, m	29.3, CH_3_	1.26, m

#### Aldehyde-α-tocopherol (**2**)

Yellow oil; [α]*^25^*_D_-1.4 (c 0.1, MeOH); UV (MeOH) λmax (log *ε*) 297 (4.32) nm; IR (film)νmax 3409, 2960, 1745, 173, 1650, 1558, 1460, 1380, 1260, 1060, 940 cm^−1^; ^1^H NMR and ^13^C NMR data, see Table 1; HRESIMS m/z 459.3425 [M-H]^-^. (calcd for C_29_H_47_O_4_, 459.3483).

#### Hydroxy-(+)-epicubenol (**6**)

White amorphous solid; [α]*^25^*_D_+42 (c 0.2, MeOH); IR (film)νmax 3340, 2968, 2896, 1460, 1381, 1058, 1012, 896 cm^−1^; ^1^H NMR and ^13^C NMR data, see Table 2; HRESIMS *m/z* 219.1745 [M+H-H_2_O]^+^ (calcd for C_15_H_23_O, 219.1702).

**TABLE 2:** ^1^H and^13^C NMR (600 MHz) data for **6-8** in CDCl_3_.

NO.	6		7		8	
	*δ*_C_, type	*δ*_H_ (*J* in Hz)	*δ*_C_, type	*δ*_H_ (J in Hz)	*δ*_C_, type	*δ*_H_ (*J* in Hz)
1	68.9, CH	4.13, d (4.8)	54.5, C	—	49.4, C	—
2	122.0, CH	5.38, d (6.0)	68.7, CH	2.90, s	50.5, CH	4.49, d (10.3)
3	128.7, C	—	79.1, CH	4.93, m	82.1, CH	4.44, d (4.5)
4	37.4, CH_2_	2.59, m, 1.96, m	51.2, CH	2.17, m	53.0, CH	2.18, m
5	46.8, CH	1.93, m	43.5, CH	2.25, m, 2.19, m	48.1, CH	3.01, d (4.3)
6	49.7, CH	2.47, dd (12.0, 3.6)	43.7, CH	2.51, m, 2.44, m	77.6, C	—
7	32.2, CH_2_	1.45, m	36.0, CH_2_	2.20, m, 1.90, m	56.2, CH_2_	3.58, m
8	32.0, CH_2_	1.71, m	30.4, CH_2_	2.34, m	64.0, CH	3.71, m
9	42.6, CH	1.75, m	104.8, C	—	79.3, CH	4.85, s
10	72.0, C	—	25.7, CH_3_	1.35, s	170.1, C	—
11	148.4, C	—	69.5, CH	3.57, d (6.0)	24.9, CH_3_	1.29, s
12	19.1, CH_3_	1.71, s	178.8, C	—	174.8, C	—
13	111.0, CH_2_	4.75, d (12.0)	24.9, CH	1.81, m	26.6, CH	2.20, m
14	15.2, CH_3_	1.12, d (6.5)	20.5, CH_3_	0.99, s	21.8, CH_3_	1.08, d (5.6)
15	23.8, CH_3_	1.72, s	21.2, CH_3_	0.98, s	20.5, CH_3_	1.01, d (6.0)
16	—	—	35.6, CH_3_	2.57, m	161.3, CH	8.25, s
17	—	—	—	—	21.4, CH_3_	2.24, s
1′	—	—	33.4, CH_2_	2.43, s	—	—
2′	—	—	175.9, C	—	—	—

#### Dendroterpene F (**7**)

White solid; [α]^25^_D_-15.9 (c 0.1, MeOH); CD (MeOH) λmax (Δε) 230 (3.15), IR (film)νmax 3440, 2950, 2862, 1780, 1590, 1460, 1383, 1240, 1040 cm^−1^; ^1^H NMR and ^13^C NMR data, see Table 2; HRESIMS *m/z* 320.1941 [M+H]^+^ (calcd for C_18_H_26_NO_4_, 320.1947).

#### Dendroterpene G (**8**)

White solid; [α]^25^_D_+12.4 (c 0.20, MeOH); CD (MeOH) λmax (Δε) 219 (3.45), IR (film)νmax 3420, 2980, 1780, 1678, 1505, 1380, 1243, 1024 cm^−1^; ^1^H NMR and ^13^C NMR data, see Table 2; HRESIMS *m/z* 354.1565 [M-H]^-^ (calcd for C_17_H_24_NO_7_, 354.1601).

### ECD calculations

The calculations of compounds (**1–2**, **7–8**) were achieved using Gaussian 16. At the B3LYP/6–311+G (d,p) level, ECD calculations were chosen for the optimized conformations. And finally, the ECD spectra were obtained by SpecDis version 1.63 software.

### Antioxidant activity assays

The DPPH radical scavenging activity was performed according to a previously described method.^30^ Briefly, a series of various concentrations of the samples (200, 100, 50, 25 μM) were mixed with DPPH (0.2 mM) in a 96-well microplate. After that, the absorbance of the mixture to react was measured at 517 nm. Percentages of the free radical-scavenging capacity of all the compounds (**1–19**) were calculated with the following equation:

$$Scavenging\;rate\left(\%\right)=\left(A_{0}-A_{1}\right)/A\times 100$$ (1)

A_0_ and A_1_ respectively represent the absorbance of the control and the samples. The ascorbic acid was the positive control in this assay. All the results were the averages of triplicate measurements. The IC_50_ values were calculated by Graphpad prism 7.0 statistic software.

### Inhibition of NO production assays

The RAW 264.7, Hela, and HepG2 cells were obtained from Southwest Medical University, and cultured in DMEM medium with 10% FBS, 2 mM glutamine, 100 U/mL of penicillin, and 100 μg/mL of streptomycin at 37°C under 5% CO2 atmosphere. The cytotoxicities of compounds (**1**–**19**) against th cell lines including RAW 264.7, Hela, and HepG2 cells were determined by the MTT method as previously reported (Lei et al., 2021). The RAW 264.7 cells were plated in six-well plates with 1×10^6^ cell/well. After 24 h, the cells were pretreated with compounds **1–19** (33 μM) for 2 h. The Griess method was used to detect NO production as previously described.

## Results and discussion

### Identification of compounds

**FIGURE 2:**
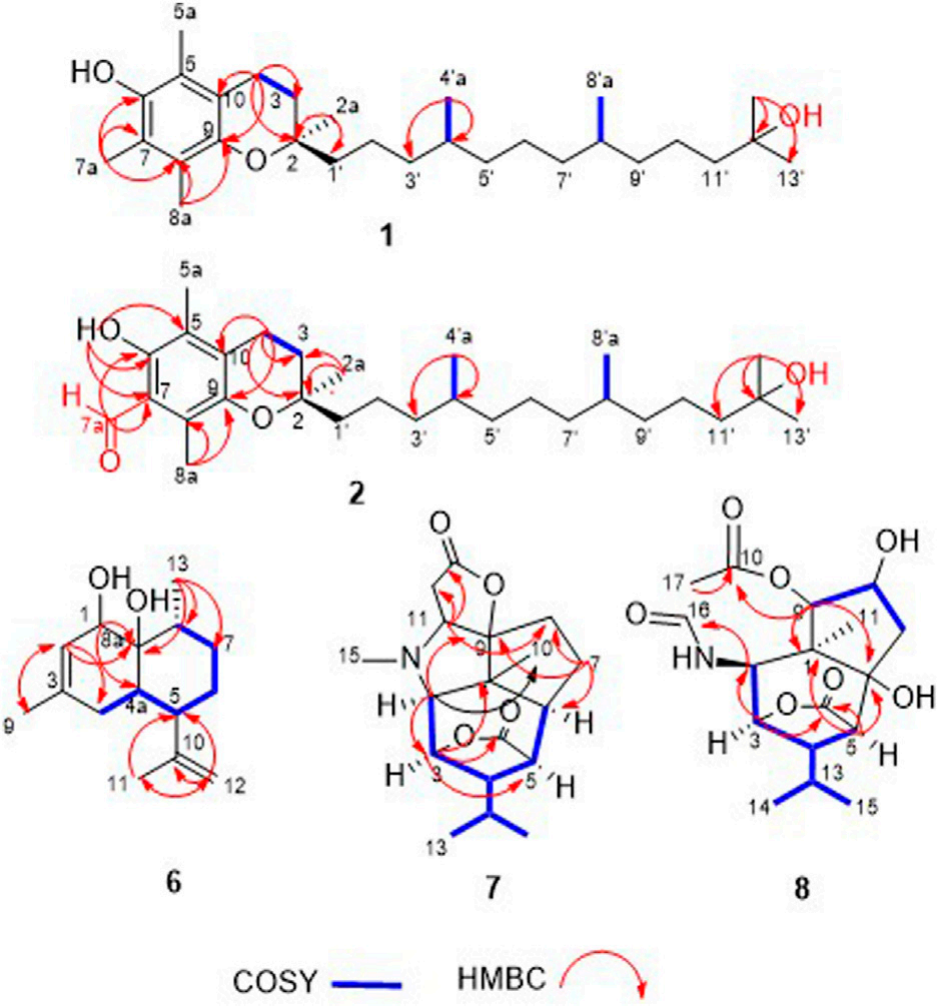
COSY and key HMBC correlations of **1–2**, **6-8**.

**FIGURE 3:**
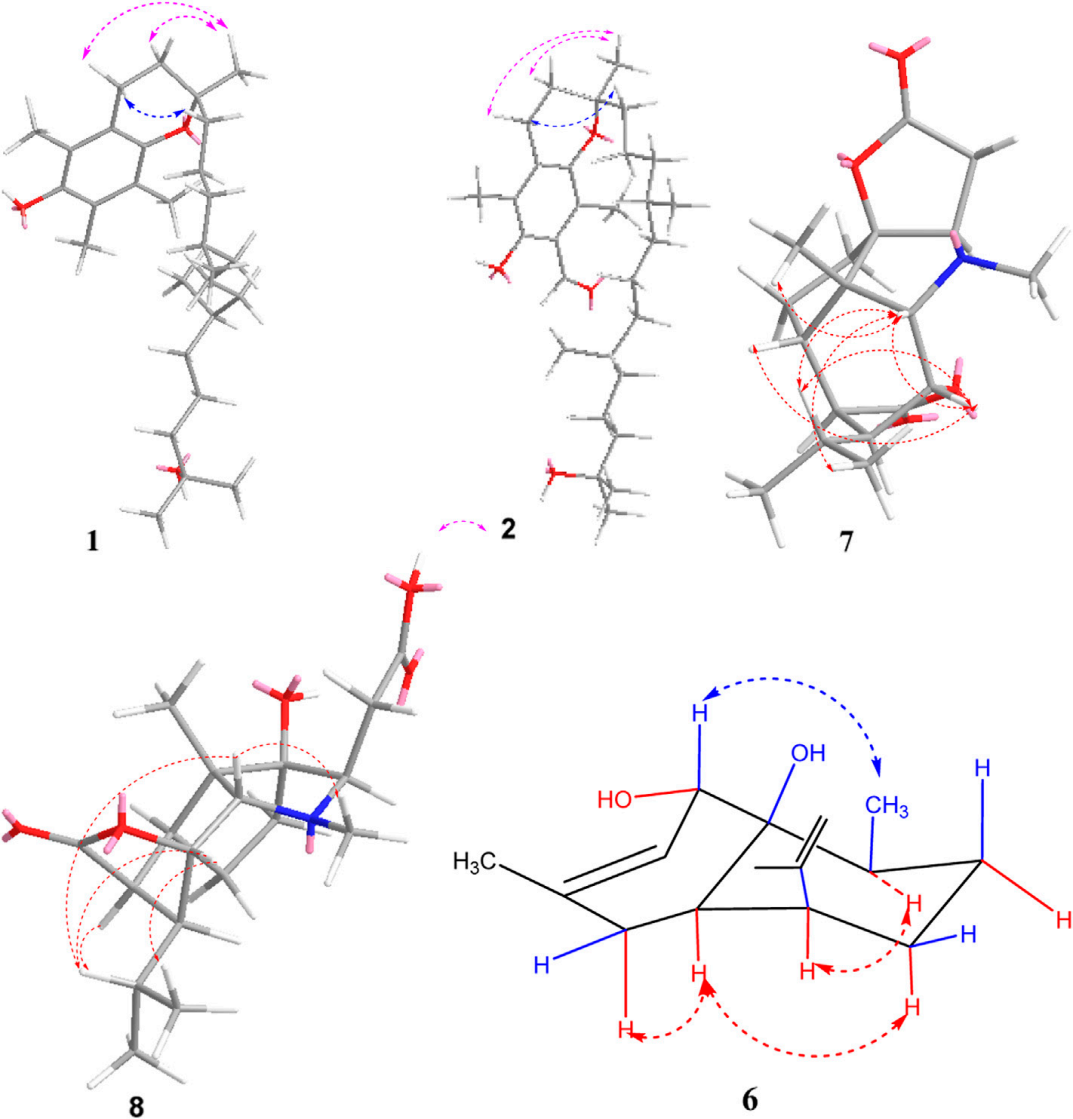
Key NOESY correlations of compounds **1–2**, **6-8**.

**FIGURE 4:**
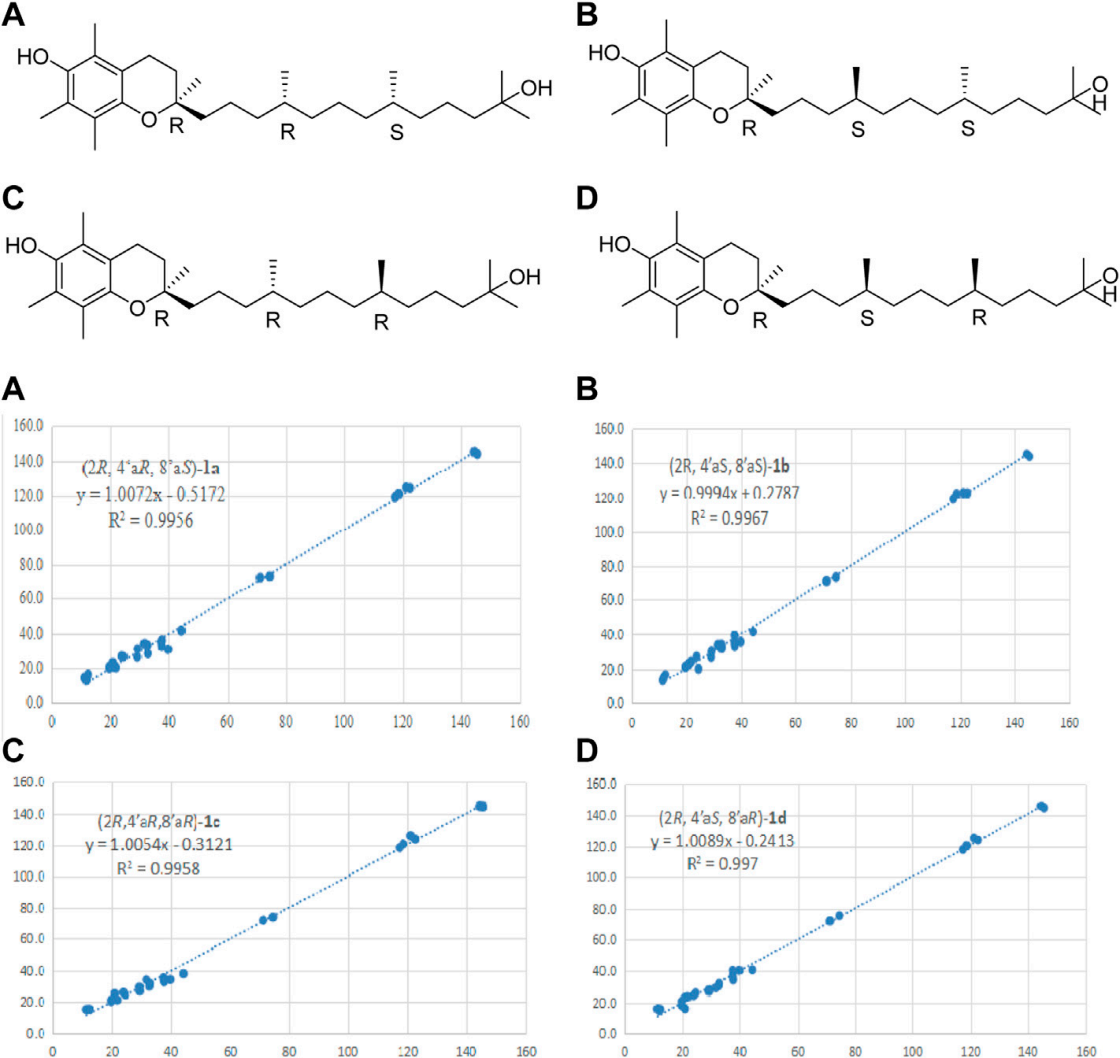
Linear regression fitting of computed ^13^C-NMR chemical shifts of the calculated configuration of compound **1** with the experimental values.

**FIGURE 5:**
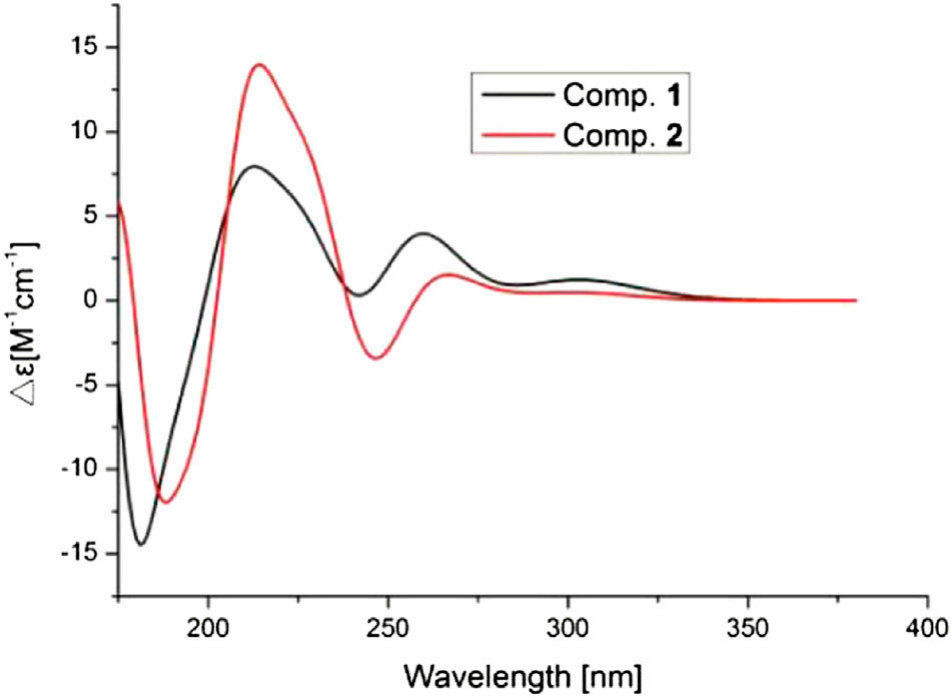
CD spectrums of **1** and **2**.

Compound **1** was obtained as a yellow oil. Its molecular formula was assigned as C_29_H_50_O_3_ by the HRESIMS at *m/z* 445.3638 [M-H]^-^. The ^1^H NMR data (see Supplementary Figure S2 in Supplementary Material) of compound **1** revealed the presence of eight methyl signals at 0.85 (d, *J* = 6.6 Hz, Me-4′a), 0.86 (d, *J* = 6.3 Hz, Me-8′a), 1.18 (m, Me-7a),1.18 (m, Me-12′a), 1.25 (m, Me-13′), 1.23 (m, Me-2a), 2.11 (s, Me-5a), 2.16 (s, Me-7a), 2.14 (s, Me-8a), two methylenes at *δ*_H_ 2.60 (t, *J* = 6.9 Hz, H-4), 1.78 (m, H-3), and other overlapped protons (Table 1). Meanwhile, the ^13^C NMR data (see Supplementary Figure S3 in Supplementary Material) showed 29 carbon signals corresponding to eight methyls, eleven methylenes, two methines carbons, and four oxygenated carbons, including two olefinic carbon, and four quaternary carbons. The similarity of NMR features of compound **1** with *α*-tocopherol suggested it was a vitamin E derivative (Fiorentino et al., 2009), with the only difference being the presence of the additional oxygenated carbons at C-12' (*δ*_C_ 71.2), which was supported by the HMBC (see Supplementary Figure S5 in Supplementary Material) correlation from H-13' (*δ*_H_ 1.25) to C-12′a (*δ*_C_ 29.2), C-11' (*δ*_C_ 44.3), and C-12' (*δ*_C_ 71.2). Furthermore, HMBC correlations between H-4 (*δ*_H_ 2.60) and C-3 (*δ*_C_ 31.6), C-2 (*δ*_C_ 74.5), C-9 (*δ*_C_ 145.5), and C-10 (*δ*_C_ 117.4) and between H-2a (*δ*_H_ 1.23) and C-1' (*δ*_C_ 39.7), C-2 (*δ*_C_ 74.5), and C-3 (*δ*_C_ 31.6) reconfirmed that the side chain located at C-2 (Figure 2). In addition, the relative stereochemistry of compound **1** was deduced based on NMR data and NOESY experiment (see Supplementary Figure S7 in Supplementary Material). The configuration of C-2, C-4′a, and C-8′a at the side chain of compound **1** was determined by comparing the ^13^C NMR data with the related compound, *α*-tocopherol and *α*-tocomonoenol (Fiorentino et al., 2009) (Kyeong-Hwa et al., 2013). The observed NOESY correlations from H-3/H-2a to H-4*α* (they were in the same plane), from H-1′*β* to H-4*β*, demonstrated that the chirality (C-2) of the compound **1** was *R* (Figure 3). In order to confirm the configuration of **1**, the calculated NMR chemical shifts of four possible diastereomers, (2*R*, 4′a*R*, 8′a*S*)-**1a**, (2*R*, 4′a*S*, 8′a*S*)-**1b**, (2*R*, 4′a*R*, 8′a*R*)-**1c**, and (2*R*, 4′a*S*, 8′a*R*)-**1d**, were obtained by DP4^+^ probability analysis at the PCM/mPW1PW91/6–31+G (d,p) level (see Supplementary Figure S42 in Supplementary Material). As shown in Figure 4, DP4^+^ analysis suggested that (2*R*, 4′a*S*, 8′a*R*)-**1d** was the most likely candidate with 98.26% probability. Meanwhile, the CD spectrum of compound **1** showed a negative Cotton effect at 183 nm and a positive Cotton effect at 208 nm suggesting the 2*R*, 4′a*S*, and 8′a*R* configuration in compound **1** (Figure 5). Hence compound **1** was determined and named 12′-Hydroxy-*α*-tocopherol.

Compound **2** was obtained as a yellow oil, of which the molecular formula was assigned to be C_29_H_48_O_4_ by ^13^C NMR data and HRESIMS *m/z* 459.3425 [M-H]^-^. It is found that the ^1^H and ^13^C NMR spectroscopic data of compound **2** (Table 1) were similar to those of compound **1**, indicating that compound **2** was also an *α*-tocopherol derivative, with the major difference being the absence of a methyl signal at C-7, while the presence of one additional aldehyde group (*δ*_H_ 10.20, *δ*_C_ 194.0) in compound **2**. This conclusion was confirmed by the HMBC (see Supplementary Figure S12 in Supplementary Material) correlations from H-7a to C-7 (*δ*_C_ 114.5), C-6 (*δ*_C_ 155.8), and C-8 (*δ*_C_ 138.4). On the basis of the similar chemical shifts and the biosynthetic pathway, we suggest that compounds **1** and **2** had the same relative configurations for C-2, C-4′a, and C-8′a. Furthermore, the observed NOESY (see Supplementary Figure S14 in Supplementary Material) correlations of compound **2** also supported the above deductions (Figure 3). The absolute configurations of compound **2** was determined by comparison of the CD spectrum. As well, the CD spectrum of compound **2** had a similar Cotton effect to those of compound **1** (Figure 5). Therefore, the structure of compound **2** was determined as 2*R*, 4′a*S*, and 8′a*R*, and named 7-aldehyde-*α*-tocopherol.

**FIGURE 6:**
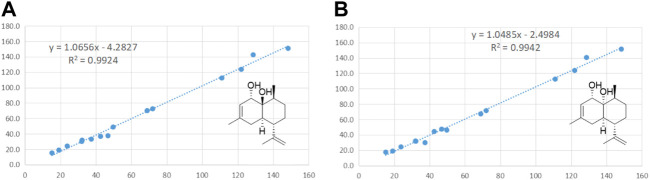
Linear regression fitting of computed ^13^C-NMR chemical shifts of the calculated configuration of compound **6** with the experimental values.

The molecular formula of compound **6** was assigned to be C_15_H_24_O_2_ on the basis of the HRESIMS spectrum at *m/z* 219.1745 [M+H-H_2_O]^+^ and ^13^C NMR data. Additionally, a detailed analysis of its ^1^H NMR data (Table 2) exhibited the existence of three olefinic protons at *δ*_H_ 5.38 (1H, s, H-2) and 4.75 (2H, d, *J* = 12.0, H-13), three methyl signals at *δ*_H_ 1.72 (3H, s, H_3_-15), 1.71 (3H, br. s, H_3_-12), and 1.12 (3H, d, *J* = 6.5, H-14). The ^13^C NMR spectrum of compound **6** exhibited 15 carbon signals, which were assigned to three methyl groups, four methylenes, four methines, two quaternary carbons, and two oxygenated carbons. The ^1^H and ^13^C NMR data of compound **6** suggested that it was very similar to a related sesquiterpene, decalin triol (Kawatsura et al., 1997), except for the presence of signals for an additional olefinic proton group (*δ*_H_ 5.38, *δ*_C_ 122.0), a methyl group (*δ*_H_ 1.72, *δ*_C_ 23.8), and the lack of signals for a hydroxy group. HMBC (see Supplementary Figure S19 in Supplementary Material) correlations between H-2 (*δ*_H_ 5.38) and C-15 (*δ*_C_ 23.8), C-4 (*δ*_C_ 37.4), and C-10 (*δ*_C_ 72.0) and between H-15 (*δ*_H_ 1.72) and C-4 (*δ*_C_ 37.4), C-2 (*δ*_C_ 122.0), and C-3 (*δ*_C_ 128.7) reconfirmed that the methyl group located at C-3. The NOESY (see Supplementary Figure S21 in Supplementary Material) correlations from H-1 to H_3_-13 and H-4α, from H-4*α* to H-5, and from H-5 to H-9 indicated that H-1, H_3_-14, H-6, and H-4α were on the same side, while H-5 and H-9 located on the opposite side (Figure 3). To further confirm the configuration of compound **6**, the theoretical NMR calculations and DP4+ probability were performed. The ^13^C NMR chemical shifts of **6a** and **6b** were calculated at the PCM/mPW1PW91/6–31+G (d,p) level. According to the DP4+ probability analyses, **6b** was the most likely candidate with 99.99% probability. Accordingly, the configuration of **6** was established (Figure 6). Therefore, compound **6** was determined to be 1-hydroxy-(+)-epicubenol.

**FIGURE 7:**
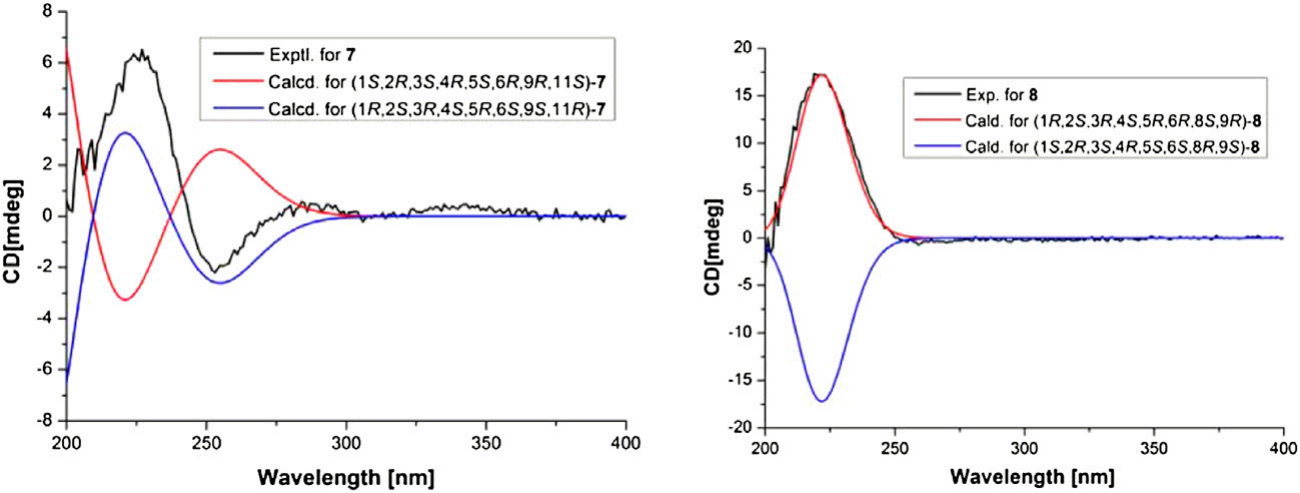
Comparison between calculated and experimental ECD spectra of **7** and **8**.

Compound **7** was obtained as a white amorphous powder. Its molecular formula, C_18_H_25_NO_4_, was established on the HRESIMS (*m/z* 320.1941 [M+H]^+^). The ^1^H NMR spectrum (Table 2) of compound **7** displayed resonances attributed to four methyl groups at *δ*_H_ 0.98 (3H, s, H_3_-15), *δ*_H_ 0.99 (3H, s, H_3_-14), *δ*_H_ 1.35 (3H, s, H_3_-10), and *δ*_H_ 2.57 (3H, m, H_3_-16), as well as an oxymethine groups at 4.93 (m, H-3). The ^13^C NMR spectrum of compound **7** displayed 18 carbon signals, which were assigned to four methyl groups, three methylenes, seven methines, four quaternary carbons, including an oxygenated quarternary carbon, two carbonyl, and an quaternary carbon center. The above data suggested that compound **7** was very similar to those of (−)-(1*R*,2*S*,3*R*,4*S*,5*R*,6*S*,9*S*,11*R*)-11-carboxymethyldendrobin (Meng et al., 2017), which was an analogue of dendrobine (Wang et al., 1985; Cassayre and Zard, 1999). The major difference was due to the presence of signals of the oxygenated quarternary carbon (*δ*_C_ 104.8) and the lactone bridge (C2′-C9) in compound **7**, instead of one methine in (−)-(1*R*,2*S*,3*R*,4*S*,5*R*,6*S*,9*S*,11*R*)-11-carboxymethyldendrobin,which was supported by the HMBC (see Supplementary Figure S26 in Supplementary Material) correlations from H-2 (*δ*_H_ 2.90) to C-9 (*δ*_C_ 104.8), C-3 (*δ*_C_ 79.1), C-11 (*δ*_C_ 69.5), C-1 (*δ*_C_ 54.5), and C-10 (*δ*_C_ 25.7), from H-7 (*δ*_H_ 1.90) to C-9 (*δ*_C_ 104.8), C-1 (*δ*_C_ 54.5), C-5 (*δ*_C_ 43.5), and C-8 (*δ*_C_ 30.4), from H-1' (*δ*_H_ 2.43) to C-2' (*δ*_C_ 175.9), C-9 (*δ*_C_ 104.8), and C-11 (*δ*_C_ 69.5) (Figure 2). In the NOESY spectrum (see Supplementary Figure S28 in Supplementary Material), the key correlations of H-2 with H_3_-14, H-10, and H-13, of H-3 with H-13, H-2, and H-6 suggested that these protons were co-facial. The relative configuration of **7** was determined as 1*R*,2*S*,3*R*,4*S*,5*R*,6*S*,11*R*. The absolute configuration of the (−)-(1*R*,2*S*,3*R*,4*S*,5*R*,6*S*,9*S*,11*R*)-11-carboxymethyldendrobin was determined using CD spectrum and synthetic product (Meng et al., 2017) (Cassayre and Zard, 1999). With comparison to the ^13^C NMR data of both compounds, compound **7** possessed the same relative configuration as the known compound. In order to determine the absolute configurations of 7, the electronic circular dichroism (ECD) spectrum of 7 was carried out. As a result, the calculated spectrum of (1*R*,2*S*,3*R*,4*S*,5*R*,6*S*,9*S*,11*R*)-7 matched well with the experimental data (Figure 7), indicating absolute configuration of 7, to be 1*R*,2*S*,3*R*,4*S*,5*R*,6*S*,9*S*,11*R*. Thus, compound **7** was established and named dendroterpene F.

The molecular formula of compound **8** was assigned to be C_17_H_25_NO_6_ on the basis of HRESIMS. And detailed analysis of the NMR data (Table 2) (see Supplementary Figure S32 in Supplementary Material) of compound **8** implied that it was very similar to those of dendroterpene B (Wang et al., 2019). However, the major difference was that one additional ester carbonyl carbon (*δ*_C_ 170.1) was present while a double bond was absent in compound **8**, indicating that the double bond was reduced to methines. And the key NOESY (see Supplementary Figure S37 in Supplementary Material) correlations of H-2 with H_3_-11, H-13, of H-3 with H_3_-15, H-13, and of H-5 with H-13 suggested the relative configuration was 1*R*, 2*S*, 3*R*, 4*S*, 5*R*, 6*R*, 8*S*, 9*R* (Figure 3). Meanwhile, the similar chemical shifts of compound **8** suggested it had the same configurations for C-1, C-2, C-3, C-4, C-5, and C-11 as dendroterpene B, the absolute configuration of which was determined by single crystal X-ray diffraction. Given that both compound **8** and dendroterpene B were isolated from the congeneric species, the absolute configuration of the former was deduced as 1*R*, 2*S*, 3*R*, 4*S*, 5*R*, 6*R*, 8*S*, and 9*R*. Then this assignment was confirmed by the calculated ECD spectrum of (1*R*, 2*S*, 3*R*, 4*S*, 5*R*, 6*R*, 8*S*, 9*R*)-**8** (Figure 7). Thus, the structure of compound **8** was finally elucidated and named dendroterpene F.

By comparing NMR data with the previous report, the structures of isolated compounds (**3**–**5**, **9**–**19**) were identified as: *α*-tocopherolquinone (**3**) (Yan et al., 2015), 2-(3,15-dihydroxy-3,7,11,15-tetramethylhexadecyl)-3,5,6-trimethyl-2,5-cyclohexadiene-1,4-dione (**4**) (Yan et al., 2015), dl-*α*-tocopherol (**5**) (Ghelf et al., 2019), mascatilin (**9**) (Majumder and Sen, 1987), stigmast-4-ene-3, 6-dione (**10**) (Shen et al., 2002), (3*β*,5*α*,20*R*,24*R*)-ster-7-en-3-ol (**11**) (Jaha et al., 1995), stigmast-4-en-6*β*-ol-3-one (**12**) (Niu et al., 2001), 7-keto-*β*-sitosterol (**13**) (Yu et al., 2007), 2, 2′-oxybis (1, 4)-di-tert-butylbenze (**14**) (Cao et al., 2020), 3-(4′-formylphenoxy)-4-methoxybenzaldehy-de (**15**) (Cao et al., 2020), (+)-syringaresinol (**16**) (Wang et al., 2009), 1,2-di-O-9Z-octadecenoyl-sn-glycero-3-phosphorylcholine (**17**) (Kwon et al., 2003), methyl 2-nonanoate-3-undecanoate-cyclohexyl-1,4-diene ester (**18**) (Li and Xu, 2018), eqi-α-amyrin (**19**) (Albert and Duilio, 2005).

#### Plausible biosynthetic pathway of compounds **7** and **8**

**SCHEME 1:**
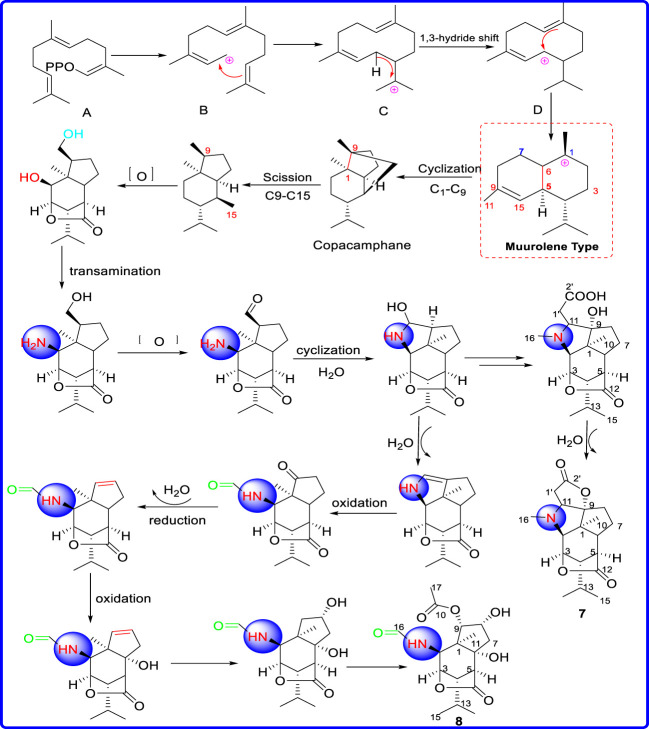
Plausible biosynthetic pathway of compounds **7** and **8**.

From a biosynthetic perspective, characterized by a picrotoxane carbon skeleton with polycyclic and seven contiguous stereocenters, dendrobines are derived from the same precursor, copacamphane. First, the copacamphane are presumably derived from farnesyl pyrophosphate (FPP) through 1,3-hydride shift, cyclization, reduction, dehydration, and oxygenation to form the core skeleton. Then copacamphane may be transformed via the break-off of carbon-carbon bonds at C (9)∼C (15) to form picrotoxane, followed by subsequent transamination, oxidation, cyclization, and methyltransferase at C (2)∼C (9) to produce compound **7**, which successively undergoes dehydration, oxidation, acetylation to provide compound **8** (Scheme 1).

### Biological activities

#### Antioxidant activity

DPPH assays are common methods to evaluate the antioxidant capacity of compounds, thus the antioxidant capacity of compounds **1**–**19** were evaluated by DPPH assays (Table 3). As a result, the DPPH radical scavenging assay showed that compound **2** (IC_50_ = 59.13 ± 2 μM) had a stronger efficiency than that of the positive control (ascorbic acid, VC) (IC_50_ = 101.67 ± 0.2 μM), while compound **19** (IC_50_ = 363.77 ± 3 μM) exhibited potent antioxidant activity as compared with the ascorbic acid.

**TABLE 3:** DPPH free-radical-scavenging of compounds **1-19** ^a^.

Sample	DPPH inhibition rate (%)	IC_50_ (μmol/L)
**1**	43.21	—
**2**	90.02	59.13 ± 2
**3**	15.88	—
**4**	18.82	—
**5**	4.19	—
**6**	10.34	—
**7**	18.62	—
**8**	8.84	—
**10**	6.22	—
**11**	36.17	—
**12**	2.78	—
**13**	2.45	—
**17**	2.45	—
**18**	2.45	—
**19**	80.36	363.77 ± 3
Ascorbic acid	90.69	101.67 ± 0.2

a Compounds that are not shown in this table did not exhibit activity.

#### Inhibition of NO production

To evaluate the activities of the isolated compounds, compounds **1–16** were tested by examining their ability to inhibit NO production in LPS-stimulated RAW 264.7 macrophage cells. The test activities showed that compounds **17** and **19** exhibited inhibition of NO production with IC_50_ values of (19.47 ± 1 μM) and (36.7 ± 2 μM), respectively (Table 4). In addition, compounds **17** and **19** did not exhibit cytotoxic. Therefore, these two compounds might serve as potential lead drug to develop NO inhibitors.

**TABLE 4:** Inhibition of NO production with IC_50_ values of compounds 17 and 19^a^.

Sample	IC_50_ (μmol/L)
**17**	36.70 ± 2
**19**	19.47 ± 1
Dexamethasone	17.46 ± 2

a Compounds that are not shown in this table did not exhibit activity.

#### Cytotoxicity activity

The cytotoxic activities of compounds (**1**–**19**) against Hela and HepG2 cells were evaluated by MTT assay (Table 5). However, Only compounds **2** and **4** exhibited cytotoxic effects against Hela cell lines with the IC_50_ values of with IC_50_ of (18.71 ± 3 μM) and (19.51 ± 1 μM), which were comparable to positive control cisplatin (14.93 ± 1 μM). In addition, compounds **2**, **3**, and **4** showed inhibitory activity against HepG2 cells with IC_50_ of (51.28 ± 3 μM), (19.75 ± 1 μM) and (37.06 ± 2 μM), respectively, and cisplatin was used as positive controls (6.58 ± 3 μM). Unfortunately, the remained compounds did not show obvious cytotoxicity.

**TABLE 5:** Cytotoxic (IC_50_ in μM) Activities of Compounds 1–4^a^.

Sample	IC_50_ (HepG2)/μM	IC_50_ (Hela)/μM
**1**	>100	—
**2**	51.28 ± 3	18.71 ± 3
**3**	19.75 ± 1	—
**4**	37.06 ± 2	19.51 ± 1
Cisplatin	6.58 ± 3	14.93 ± 1

a Compounds that are not shown in this table did not exhibit activity.

In addition, comparison of the structural characteristics among these compounds, the structure activity relationship is discussed. Compound **2** displayed higher antioxidant activity than compound **1**, which may be due to the additional aldehyde group at C-7 in **2**, which was in agreement with a previous report (Fiorentino et al., 2009). This result might indicate that the aldehyde group is an important functional group that can increase antioxidant capacity. Comparing with the antioxidant activity compound **2**, compound **3** was inactive, which indicated the side chain group at C-2 for the compounds **2** and **3**, were not essential for antioxidant activities.

## Conclusion

As a popular cash crop and a traditional herbal medicine, the extract of *D. nobile* Lindl. has been reported to possess antioxidant, anti-tumour, and anti-inflammatory activities. However, the chemical constituents and biological activities of *Dendrobium nobile* have not been elucidated yet. Therefore, in our current study, chemical composition of the extract of *Dendrobium nobile* was isolated and analyzed. Nineteen compounds, including five new compounds (**1–2**, **6–8**) and fourteen known compounds (**3–5**, **9–19**). Antioxidant tests indicated that compound **2** displayed significant antioxidant activity, while compound **19** displayed weak antioxidant activity compared with ascorbic acid (VC). Meanwhile, compound **2** possessed the aldehyde group, which might be an important functional group for antioxidant ability. Besides, the structure-activity relationships (SARs) of tested compounds also suggested that the aldehyde group may play an important role in DPPH radical scavenging. These results implied that the structural variation of vitamin E analogues significantly affected the bioactivity. In addition, compounds **2** and **4** also exhibited an equal effect to positive control cisplatin. Therefore, this research provided a reliable support for *Dendrobium nobile* as potential application in the pharmaceutical and food industries.

Additionally, increasing evidence suggests that oxidative stress plays an important role in the pathogenesis of Alzheimer’s disease (AD) (Guidi et al., 2006). Thus, the inhibition of oxidative stress could be a feasible treatment against Alzheimer’s disease (AD). Fortunately, studies have shown that natural antioxidants could exert positive effects against oxidative stress-induced chronic metabolic diseases (Xu et al., 2015). As an important source of natural antioxidants, the *Dendrobium nobile* has attracted much attention in the treatment of AD (Yan et al., 2015). Interestingly, compound **19** not only exhibited antioxidant activity, but also displayed inhibition of NO production, while did not exhibit cytotoxic. The result is consistent with the results reported in the previous literature (Nie et al., 2020). Antioxidant and anti-inflammatory activities are associated with a series of signaling pathways, which may interact with each other in the pathological process of AD (Nie et al., 2020). Therefore, compound **19** might serve as potential lead drug to treat Alzheimer’s disease (AD). Findings from this current study provide insights on the phytochemical constituents and significant antioxidant activities of *Dendrobium nobile*, which are critical for the further development for its applications in Alzheimer’s disease (AD).

## Data Availability

The original contributions presented in the study are included in the article/Supplementary Material, further inquiries can be directed to the corresponding authors.
