# Supplementary material for: Antioxidant and anti-inflammatory activity of constituents isolated from Dendrobium nobile (Lindl.)
Source: Front Chem. 2022 Oct 4;10:988459. doi: 10.3389/fchem.2022.988459 (PMC9577000; doi:10.3389/fchem.2022.988459)
Supplement: Supplementary file 1 [file DataSheet1.docx]

Supporting Information

**Antioxidant and anti-inflammatory activity of constituents isolated from *Dendrobium nobile* (Lindl.)**

**Hui Lei ^1,#^, Shunmei Zou ^1,#^, Jiafu Lin ^3^, Longfei Zhai ^4^, Yifeng Zhang ^1^, Xiujuan Fu ^1^, Siwei Chen ^1^, Hong Niu ^1^, Feifei Liu ^5^, Chunlian Wu ^2^ *, Dan Zhang ^1^ ***

1. *School of Pharmacy, Southwest Medical University, Luzhou 646000, P. R.* [*China; huilei@swmu.edu.cn;*](mailto:China;huilei@swmu.edu.cn;) [*fuxiujuan2021@swmu.edu.cn; dawn123454@swmu.edu.cn;*](mailto:fuxiujuan2021@swmu.edu.cn,dawn123454@swmu.edu.cn,) [*chensiwei2021@swmu.edu.cn;*](mailto:chensiwei2021@swmu.edu.cn,) [*uoyu@163.com; 15550325896@163.com;*](mailto:uoyu@163.com;15550325896@163.com;) *zsm03131820@163.com*
2. *Key Laboratory of Southwest China Wildlife Resources Conservation (China West Normal University), Ministry of Education, Nanchong, Sichuan 637009, P. R. China; wcl_xj@163.com;*
3. *School of Pharmacy, Chengdu University, Chengdu, Sichuan 610052, P.R. China; linjiafu@cdu.edu.cn;*
4. *Antibiotics Research and Re-evaluation Key Laboratory of Sichuan Province, Sichuan Industrial Institute of Antibiotics, Chengdu University, Chengdu, Sichuan 610052, P.R. China; NPR2008@163.com;*
5. *School of Life Sciences, Jiangsu Normal University, Xuzhou, Jiangsu, 221116, China*

**Corresponding author.**

E-mail addresses: zhangdan@swmu.edu.cn (D. Zhang)

E-mail addresses: leihui-2008@163.com

Figure S1. HRESI-MS spectrum of the new compound **1**

Figure S2. ^1^H NMR (600 MHz, CDCl_3_) spectrum of the new compound **1**

Figure S3. ^13^C NMR (150 MHz, CDCl_3_) spectrum of the new compound **1**

Figure S4. HSQC spectrum of the new compound **1**

Figure S5. HMBC spectrum of the new compound **1**

Figure S6. COSY spectrum of the new compound **1**

Figure S7. NOESY spectrum of the new compound **1**

Figure S8. HRESI-MS spectrum of the new compound **2**

Figure S9. ^1^H NMR (600 MHz, CDCl_3_) spectrum of the new compound **2**

Figure S10. ^13^C NMR (150 MHz, CDCl_3_) spectrum of the new compound **2**

Figure S11. HSQC spectrum of the new compound **2**

Figure S12. HMBC spectrum of the new compound **2**

Figure S13. COSY spectrum of the new compound **2**

Figure S14. NOESY spectrum of the new compound **2**

Figure S15. HRESI-MS spectrum of the new compound **6**

Figure S16. ^1^H NMR (600 MHz, CDCl_3_) spectrum of the new compound **6**

Figure S17. ^13^C NMR (150 MHz, CDCl_3_) spectrum of the new compound **6**

Figure S18. HSQC spectrum of the new compound **6**

Figure S19. HMBC spectrum of the new compound **6**

Figure S20. COSY spectrum of the new compound **6**

Figure S21. NOESY spectrum of the new compound **6**

Figure S22. HRESI-MS spectrum of the new compound **7**

Figure S23. ^1^H NMR (600 MHz, CDCl_3_) spectrum of the new compound **7**

Figure S24. ^13^C NMR (150 MHz, CDCl_3_) spectrum of the new compound **7**

Figure S25. HSQC spectrum of the new compound **7**

Figure S26. HMBC spectrum of the new compound **7**

Figure S27. COSY spectrum of the new compound **7**

Figure S28. NOESY spectrum of the new compound **7**

Figure S29. ^1^H NMR (DMSO-*d*_6_) spectrum of the new compound **7**

Figure S30. NOESY (DMSO-*d*_6_) spectrum of the new compound **7**

Figure S31. HRESI-MS spectrum of the new compound **8**

Figure S32. ^1^H NMR (600 MHz, CDCl_3_) spectrum of the new compound **8**

Figure S33. ^13^C NMR (150 MHz, CDCl_3_) spectrum of the new compound **8**

Figure S34. HSQC spectrum of the new compound **8**

Figure S35. HMBC spectrum of the new compound **8**

Figure S36. COSY spectrum of the new compound **8**

Figure S37. NOESY spectrum of the new compound **8**

Figure S38. The data of DP4plus method of compound **1**

Figure S39. Comparison of the experimental ^13^C NMR data and mean absolute error of compound **1**

Figure S40. The data of DP4plus method of compound **6**

Figure S41. Comparison of the experimental ^13^C NMR data and mean absolute error of compound **6**

Figure S42. DP4^+^ probability of ^13^C NMR chemical shifts（compound **1** and **6**）.

Figure S43. Statistics of Ordinary Least Squares (OLS) Linear Regression of experimental and computed ^13^C-NMR chemical shifts.

# Energies and Populations

## Energies at M062X/6-31G(d) theory level (Table S1-S6)


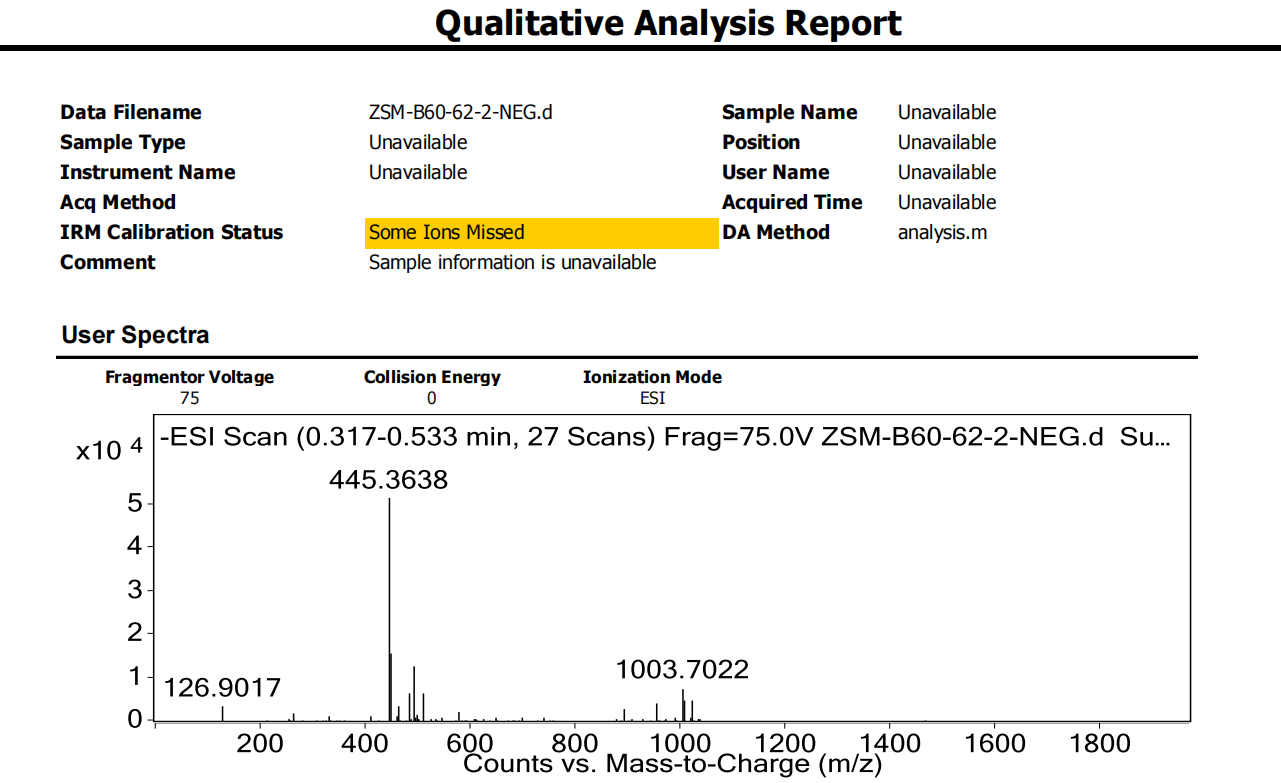


Figure S1. HRESI-MS spectrum of the new compound **1**


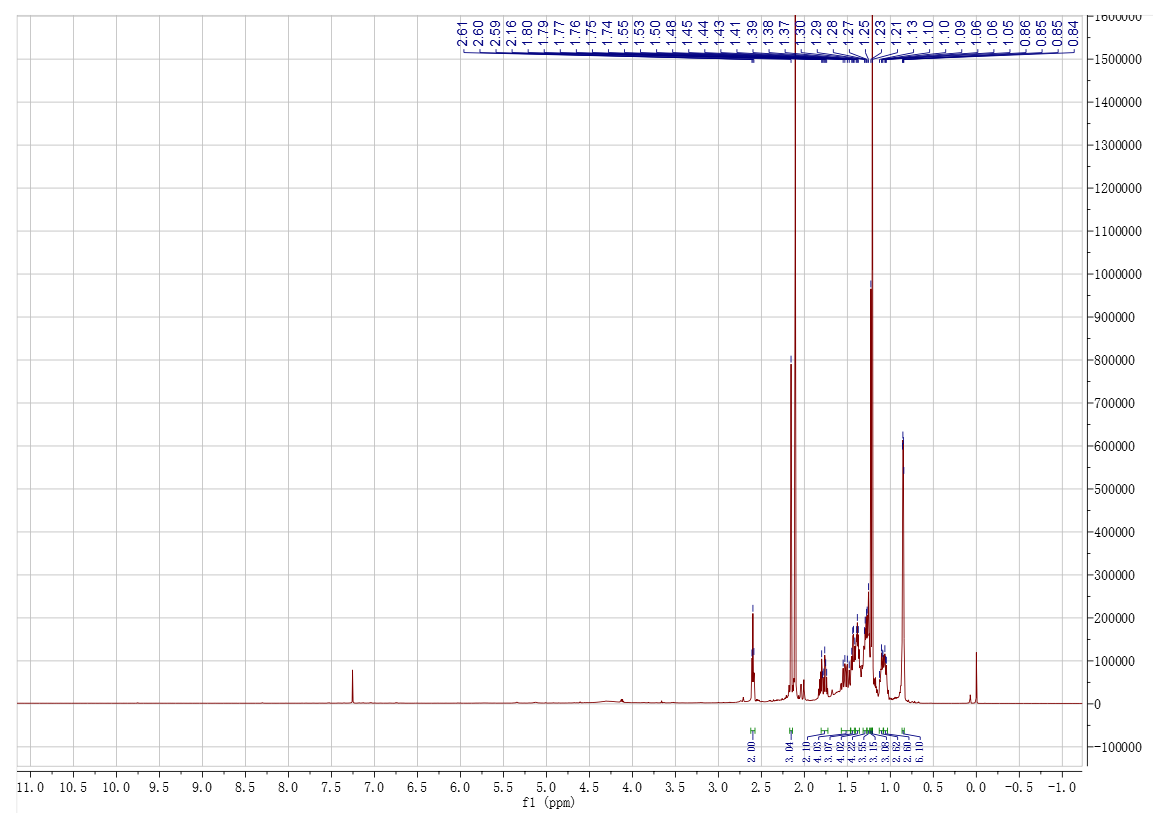


Figure S2. ^1^H NMR (600 MHz, CD_3_OD) spectrum of the new compound **1**


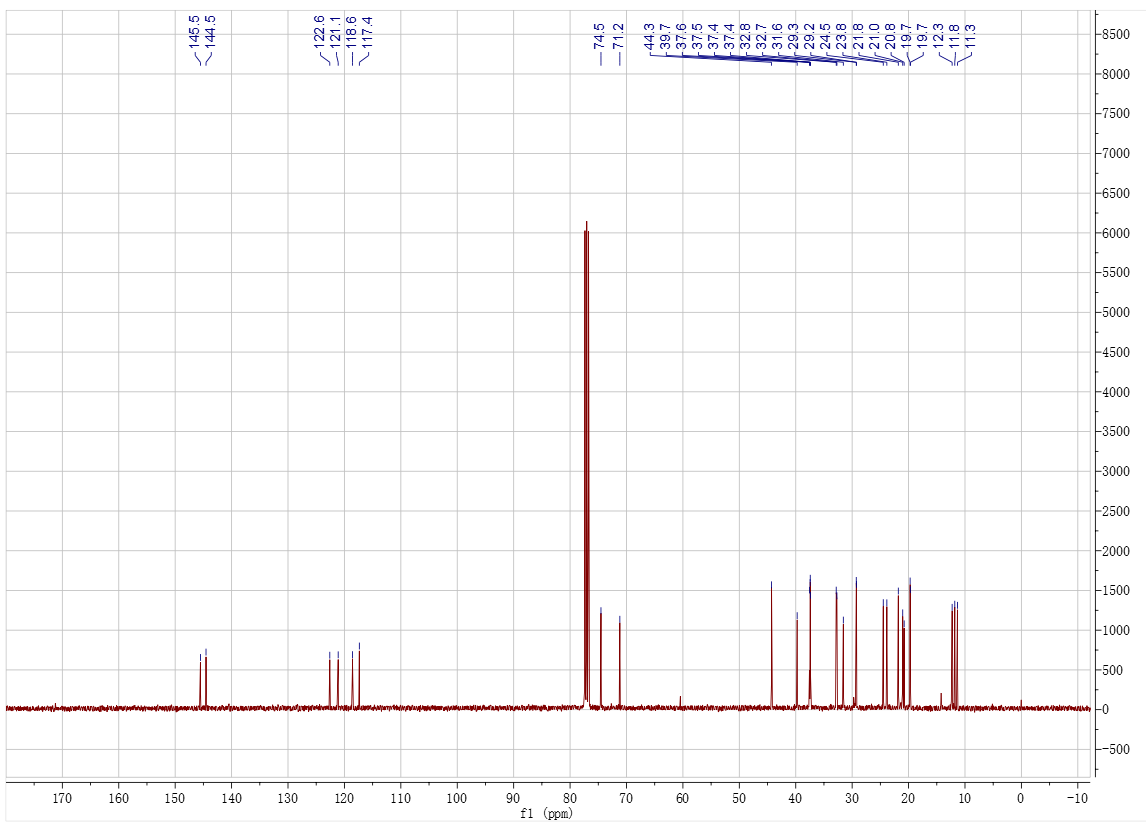


Figure S3. ^13^C NMR (150 MHz, CD_3_OD) spectrum of the new compound **1**


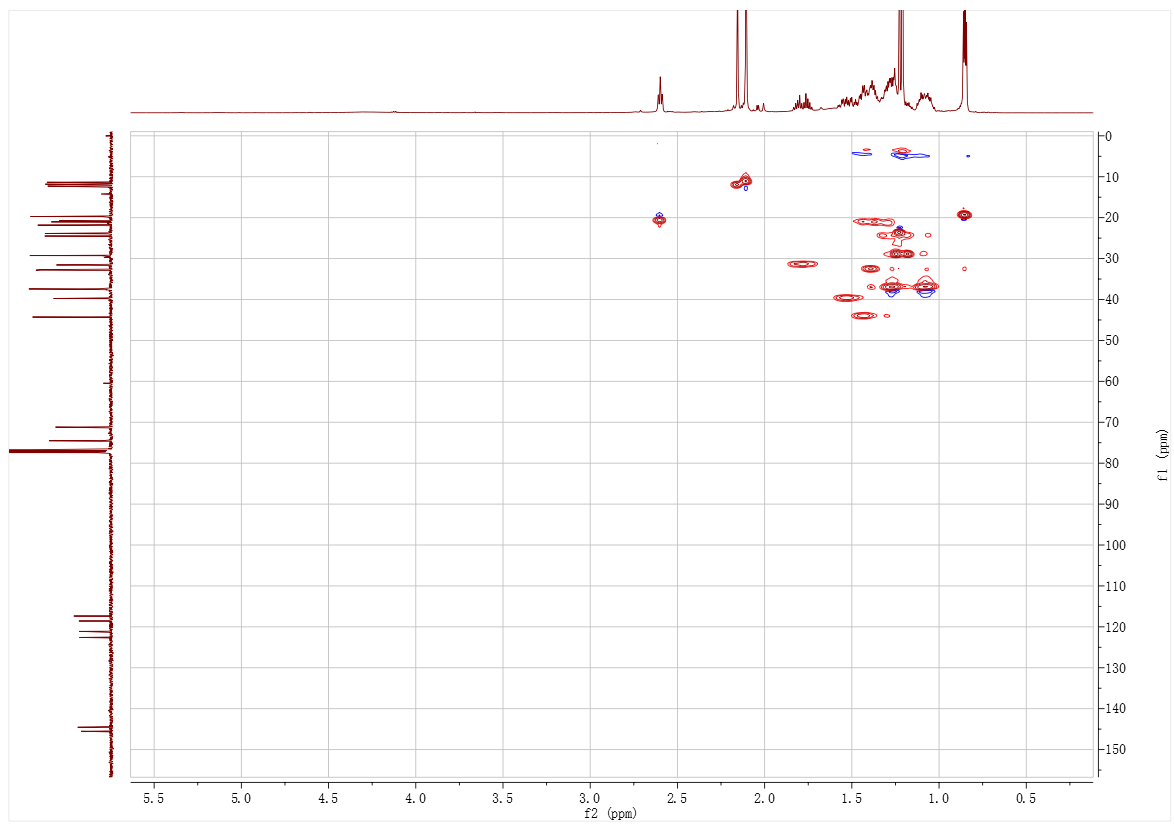


Figure S4. HSQC spectrum of the new compound **1**


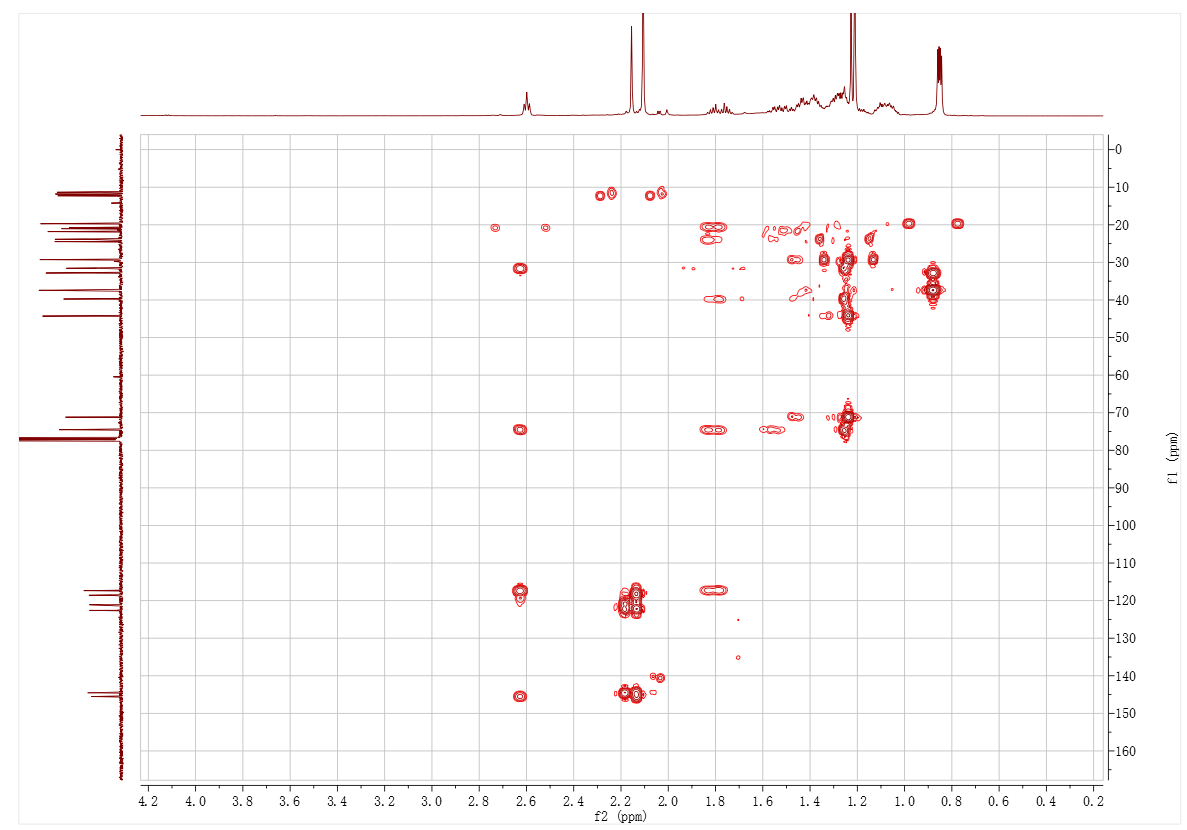


Figure S5. HMBC spectrum of the new compound **1**


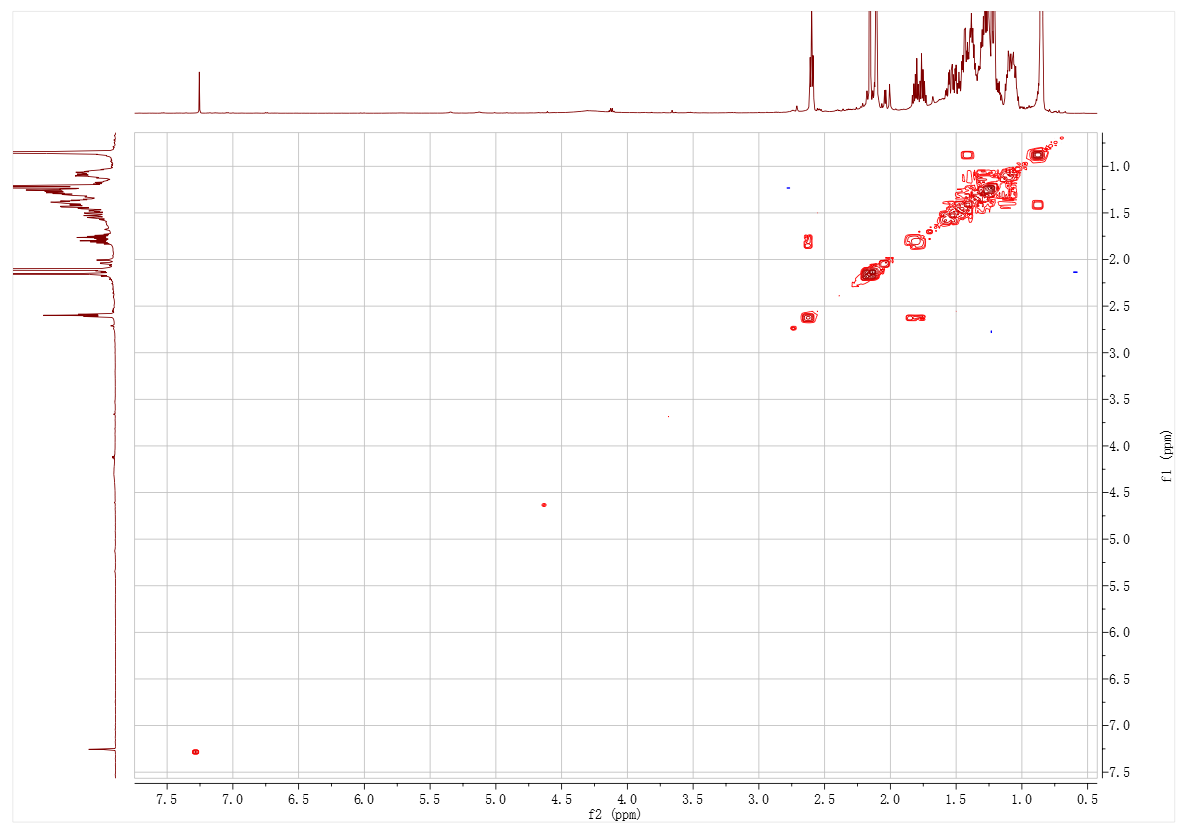


Figure S6. COSY spectrum of the new compound **1**


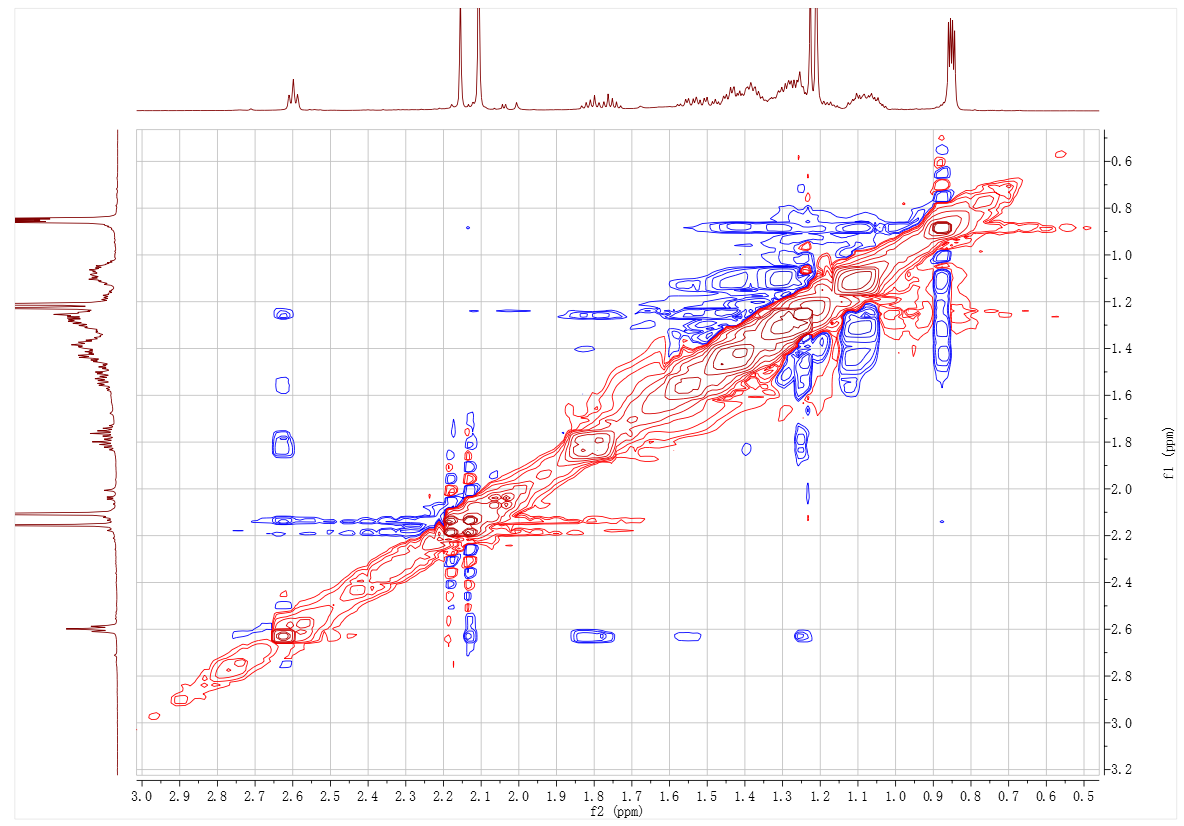


Figure S7. NOESY spectrum of the new compound **1**


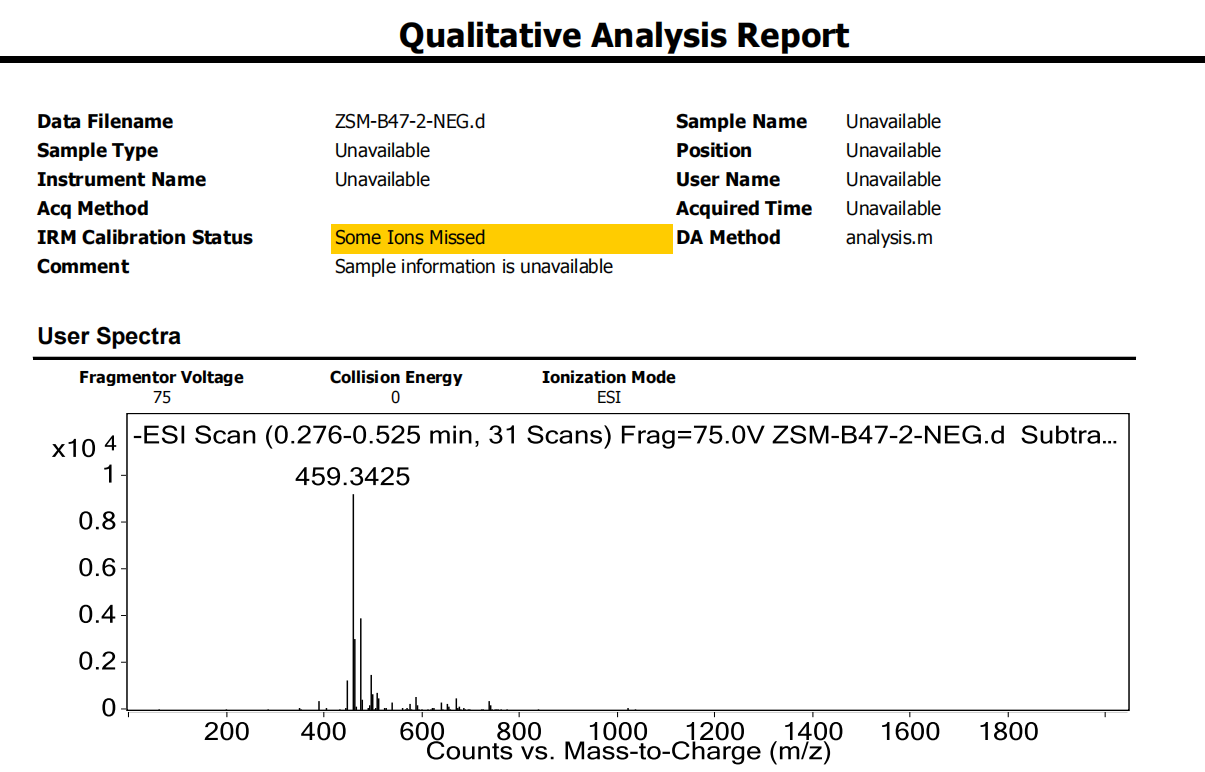


Figure S8. HRESI-MS spectrum of the new compound **2**


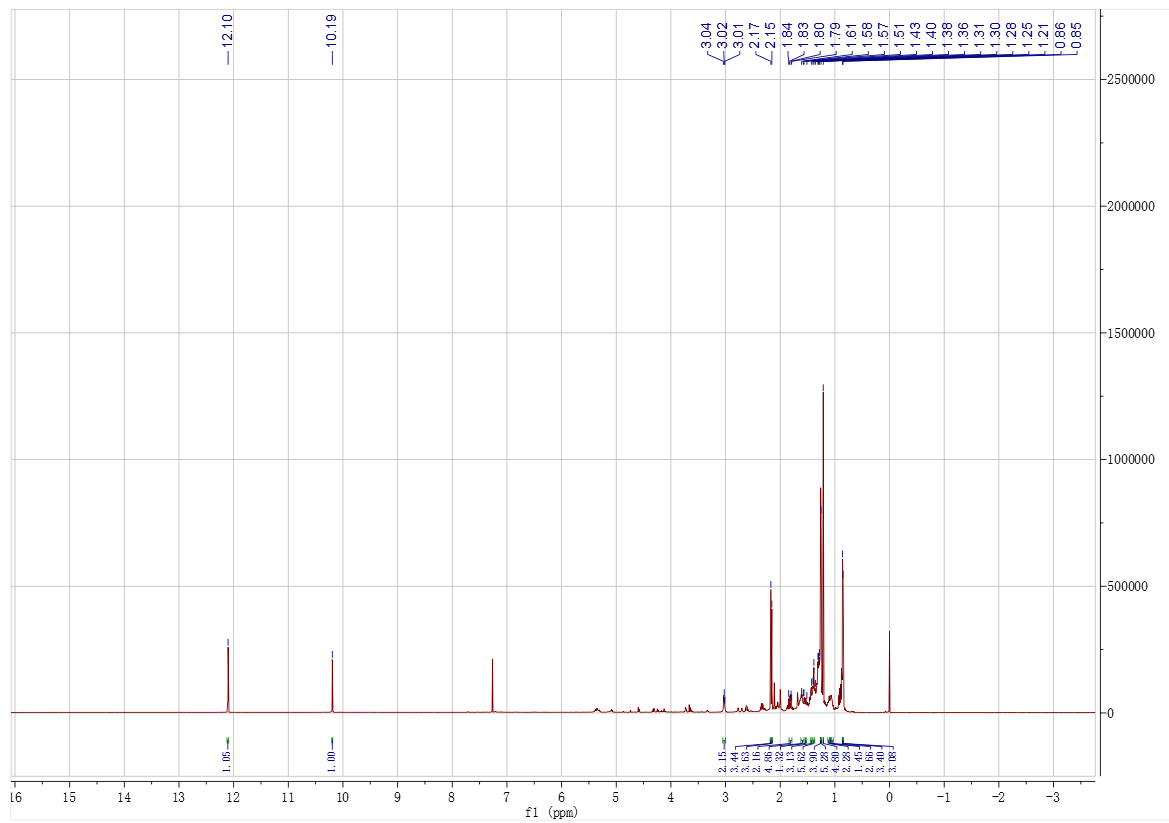


Figure S9. ^1^H NMR (600 MHz, CD_3_OD) spectrum of the new compound **2**


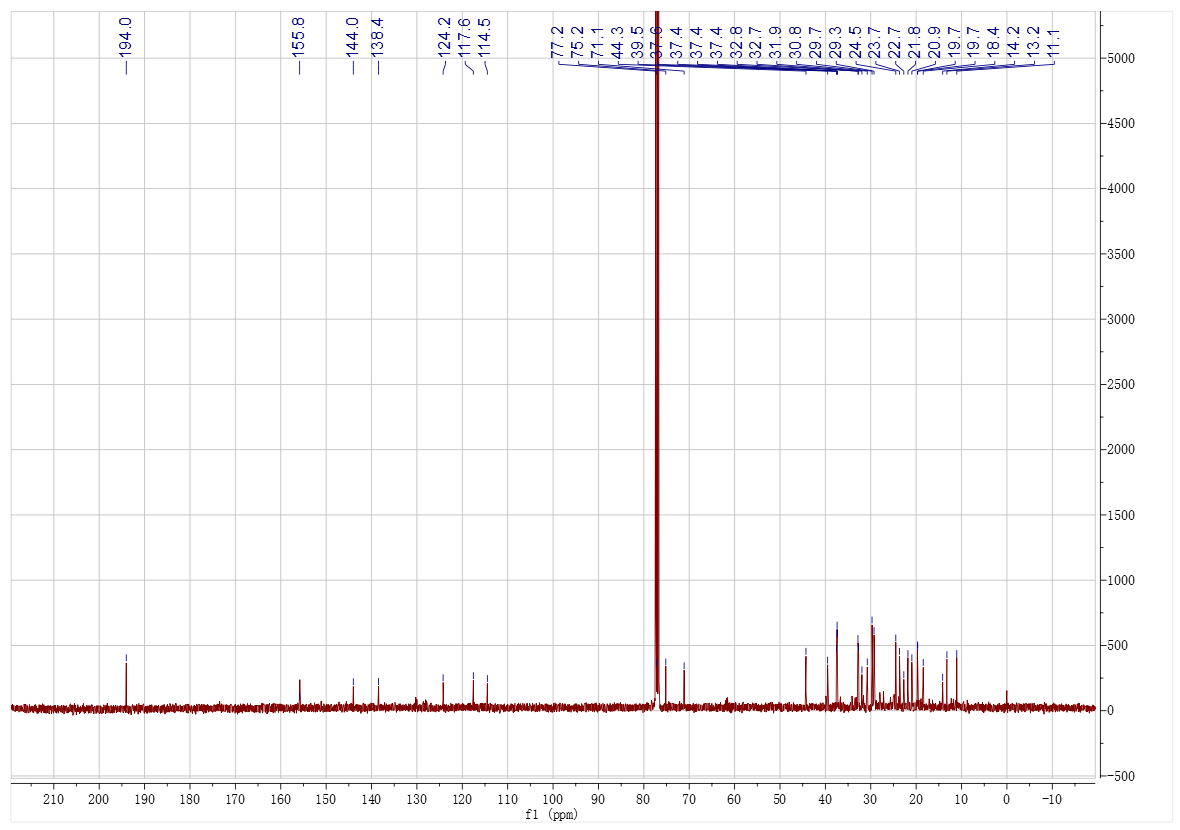


Figure S10. ^13^C NMR (150 MHz, CD_3_OD) spectrum of the newcompound **2**


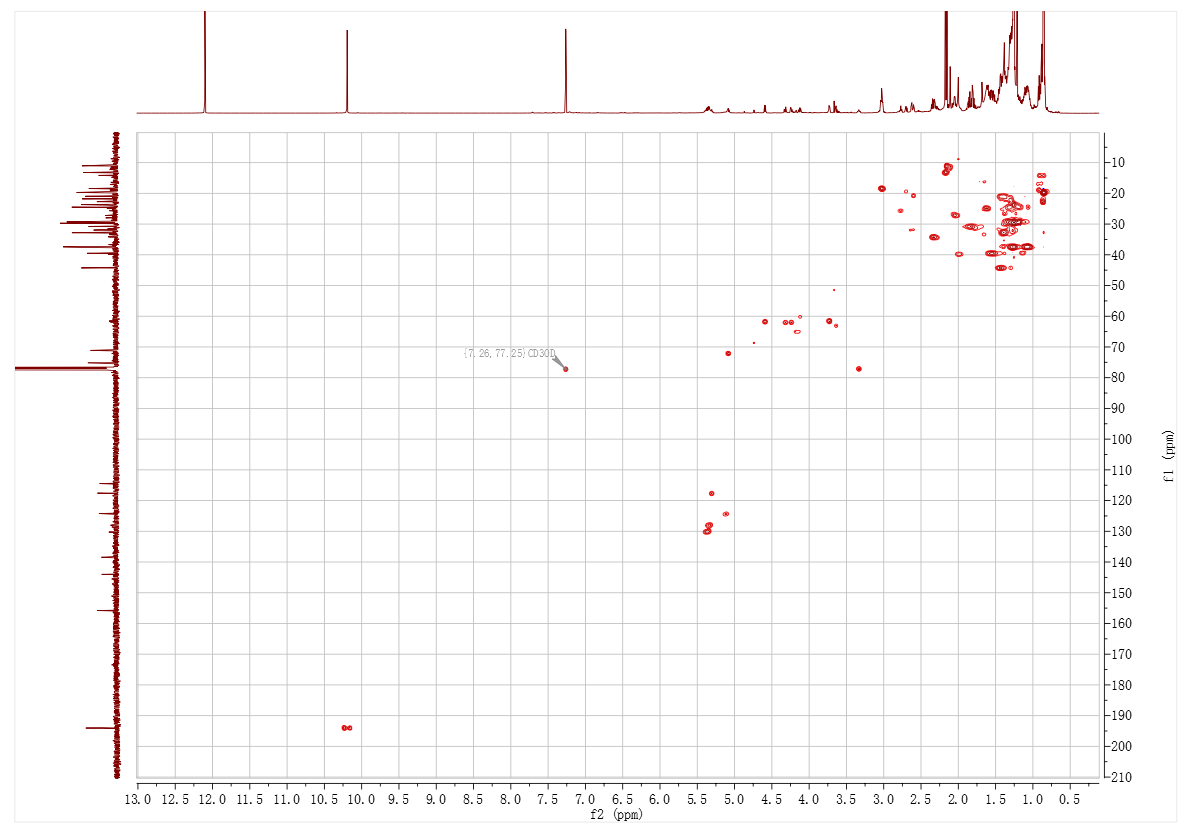


Figure S11. HSQC spectrum of the new compound **2**


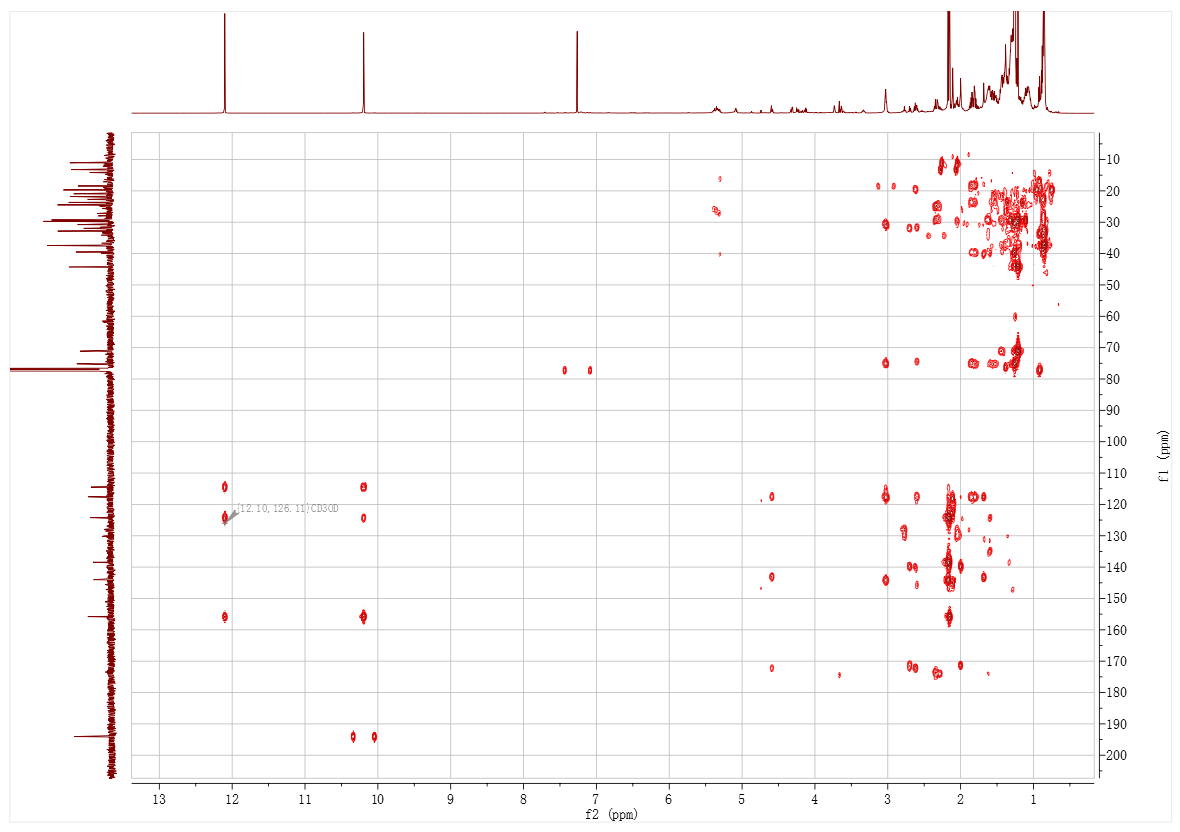


Figure S12. HMBC spectrum of the new compound **2**


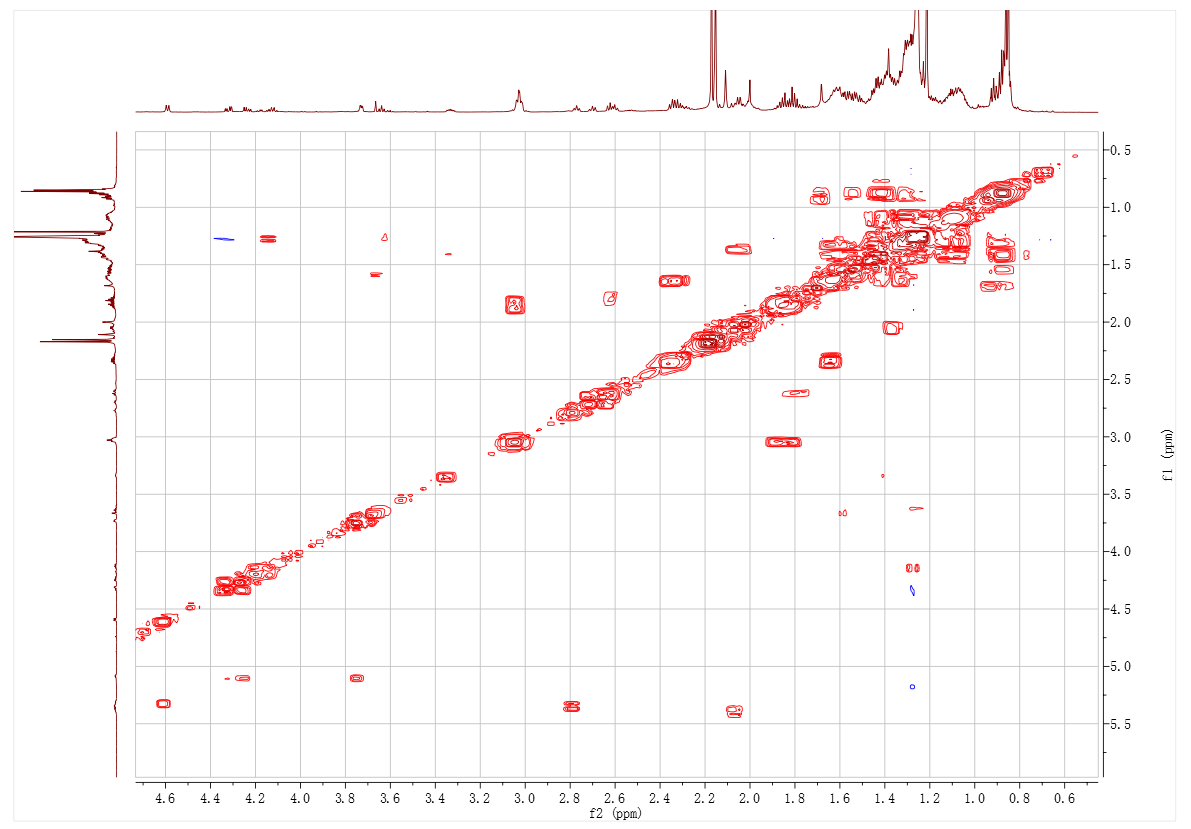


Figure S13. COSY spectrum of the new compound **2**


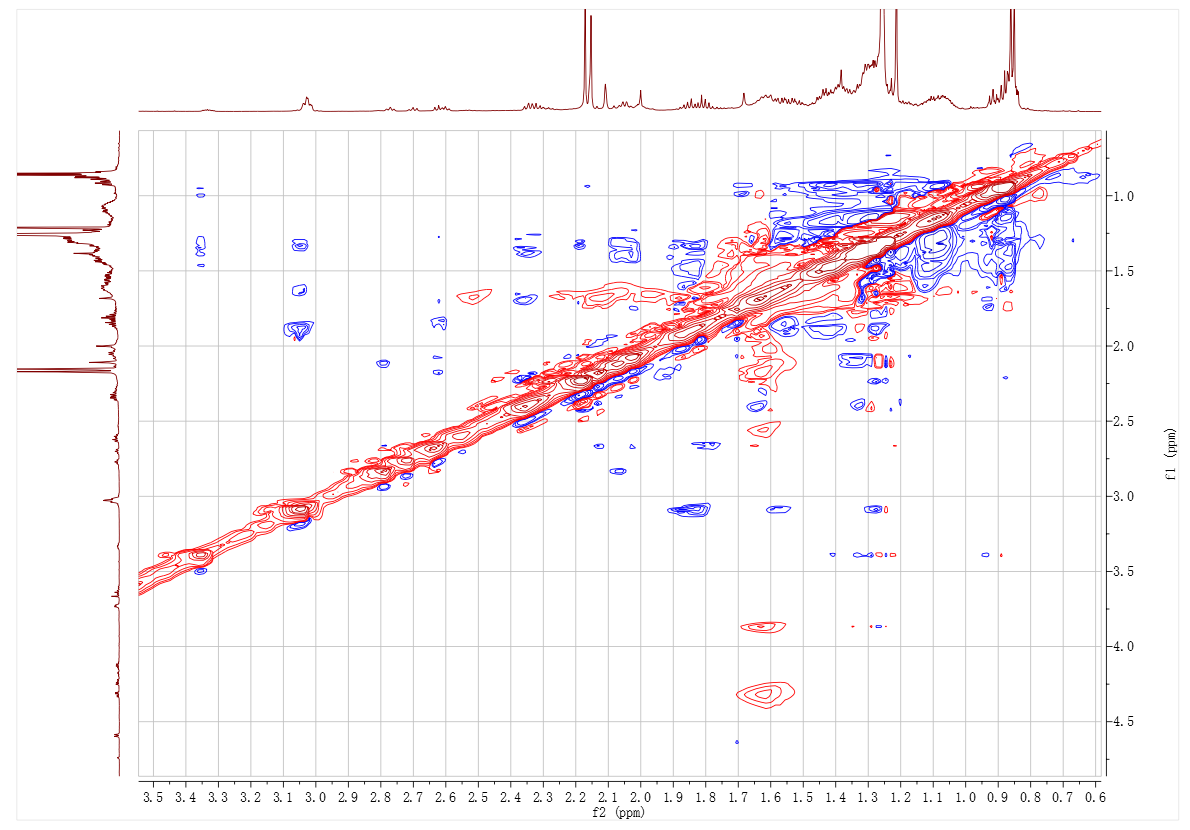


Figure S14. NOESY spectrum of the new compound **2**


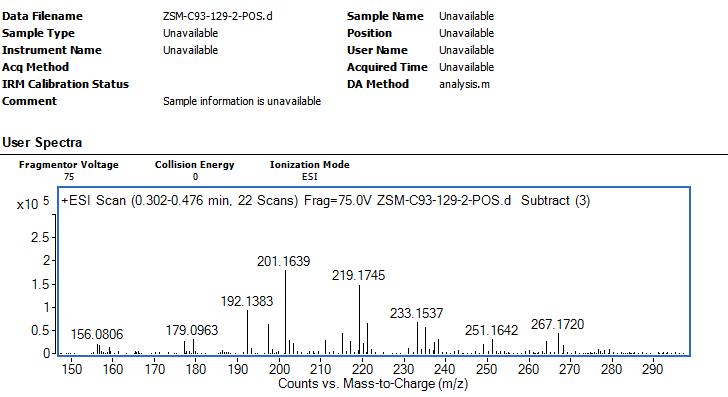


Figure S15. HRESI-MS spectrum of the new compound **6**


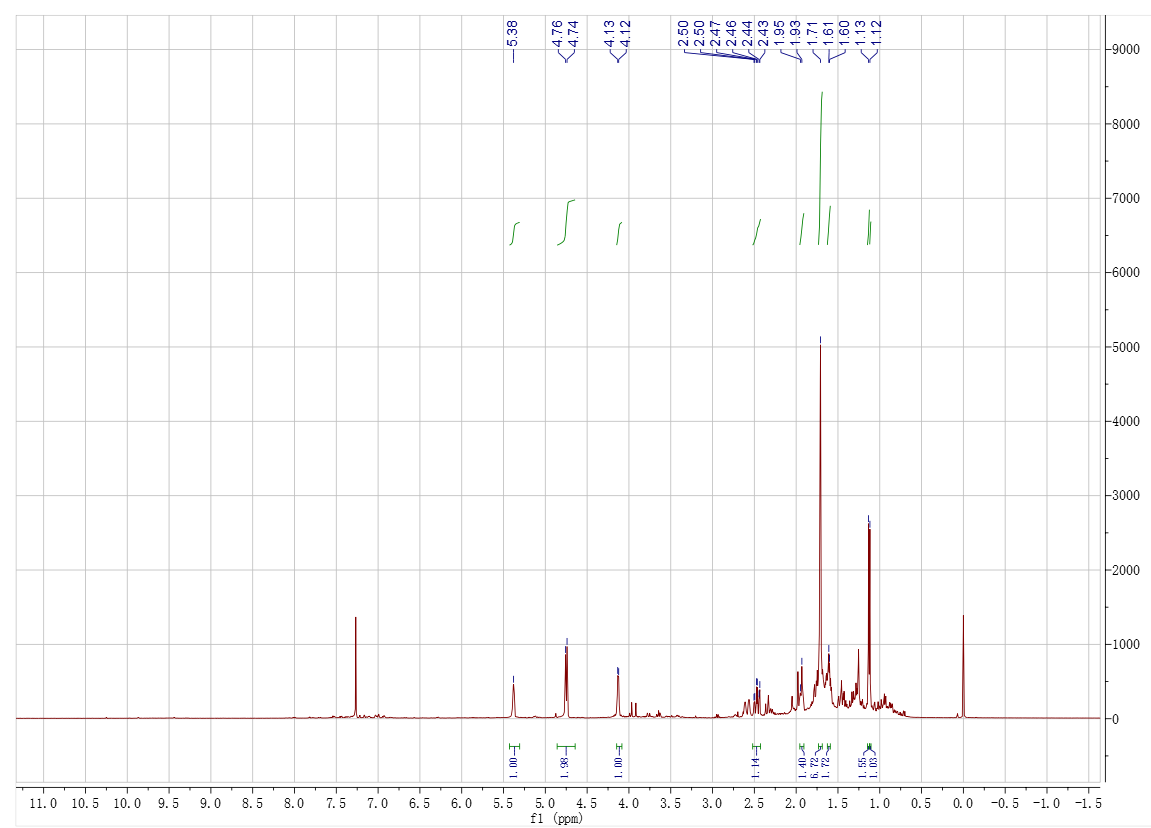


Figure S16. ^1^H NMR (600 MHz, CD_3_OD) spectrum of the new compound **6**


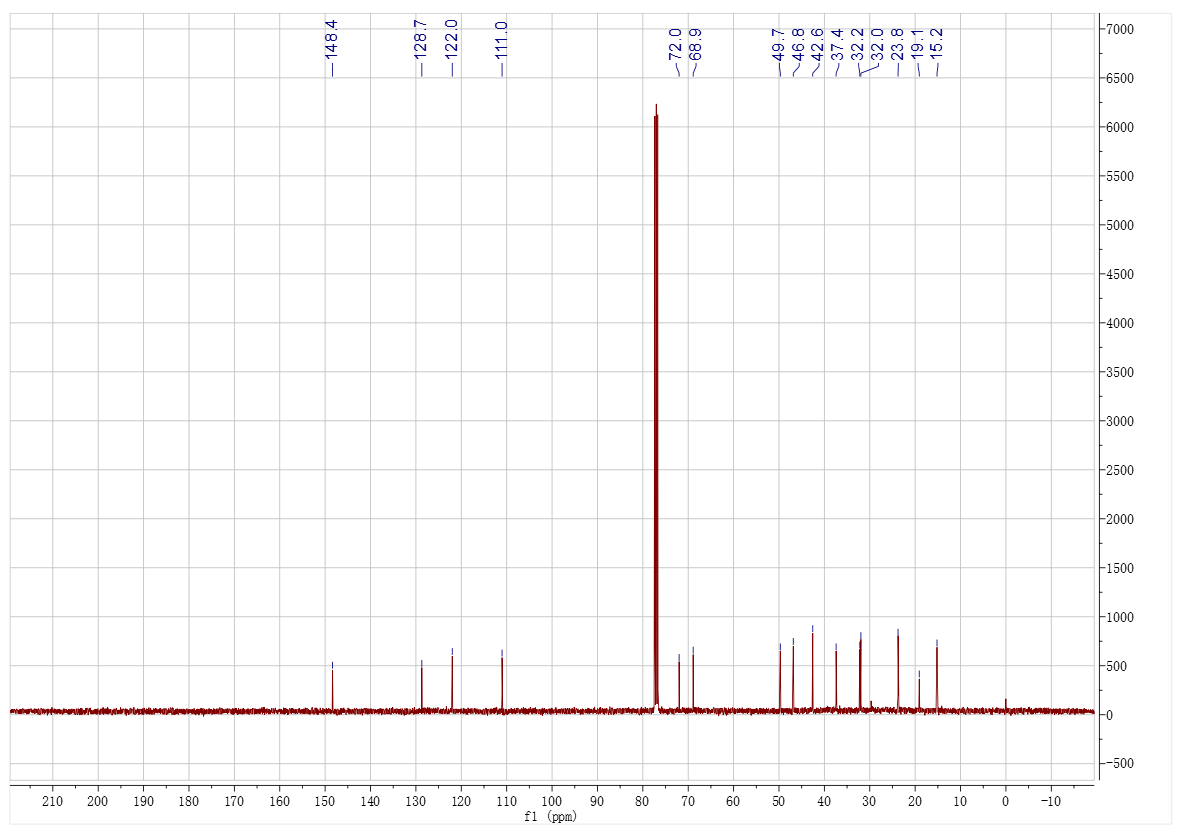


Fiure S17. ^13^C NMR (150 MHz, CD_3_OD) spectrum of the new compound **6**


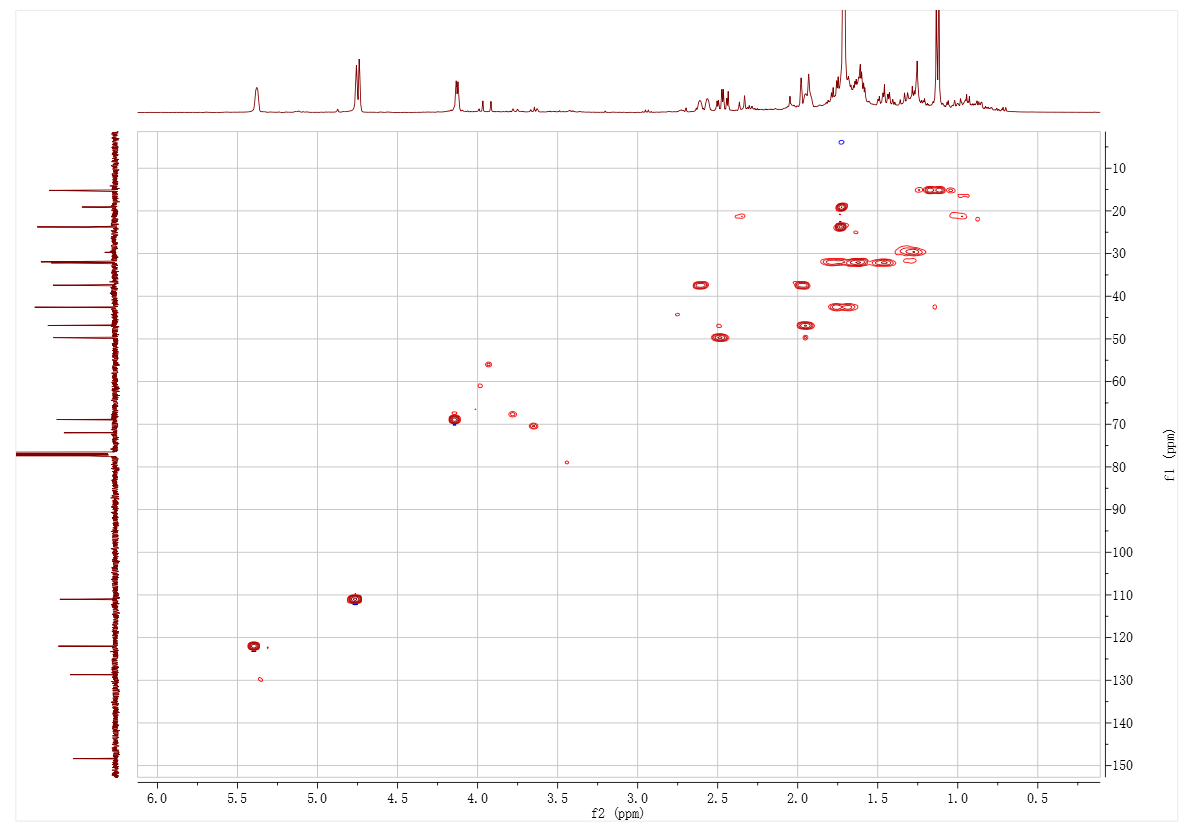


Figure S18. HSQC spectrum of the new compound **6**


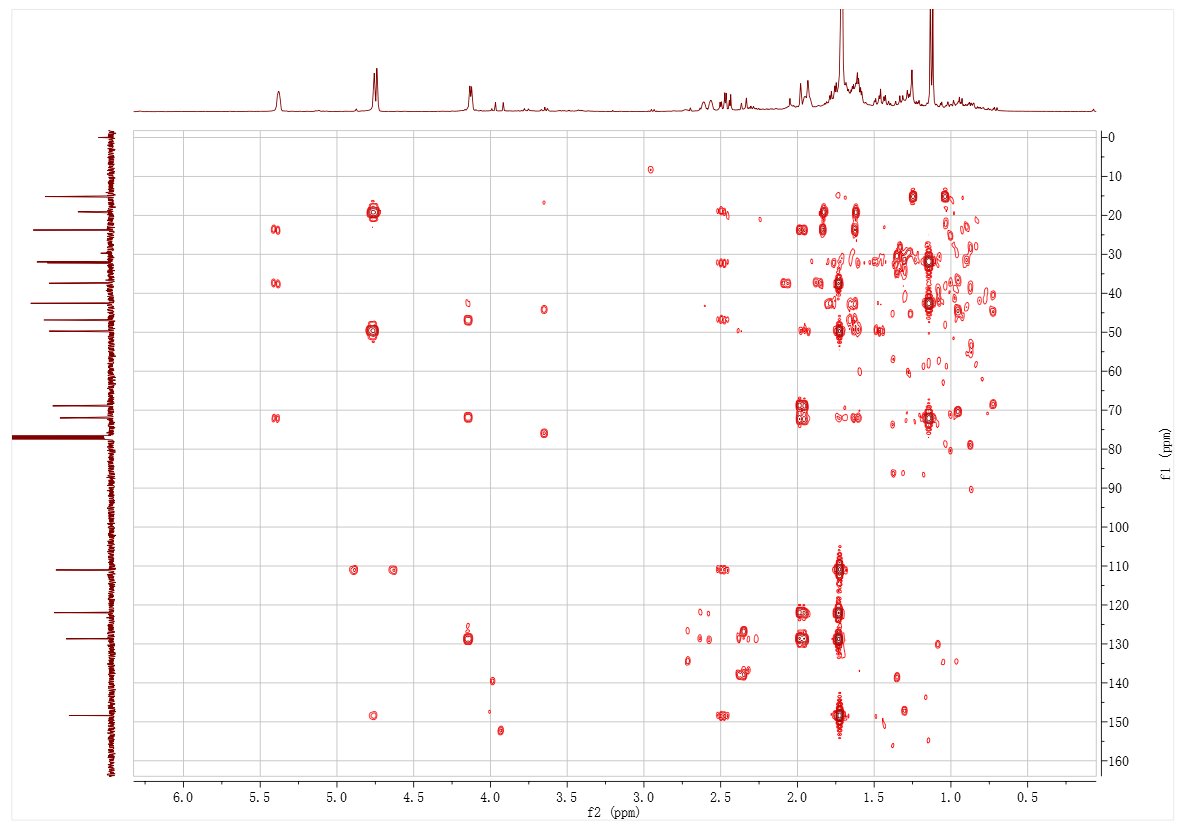


Figure S19. HMBC spectrum of the new compound **6**


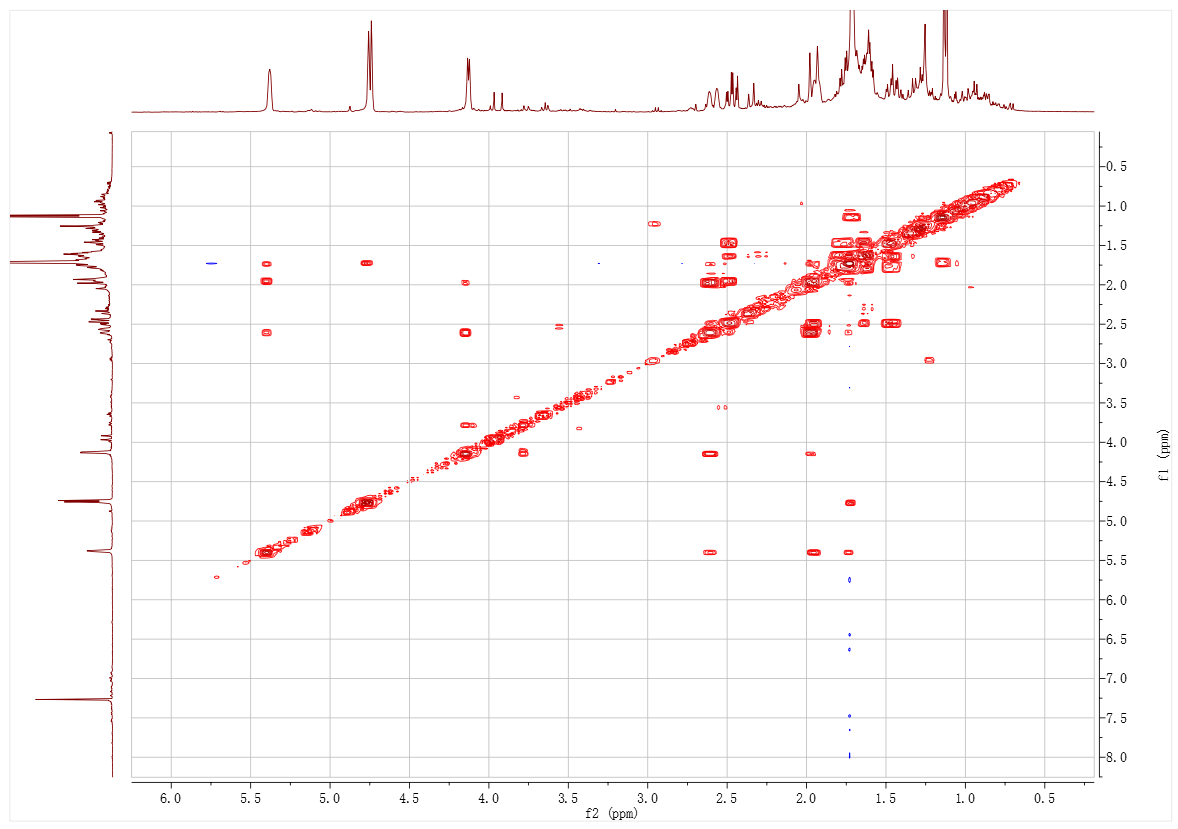


Figure S20. COSY spectrum of the new compound **2**


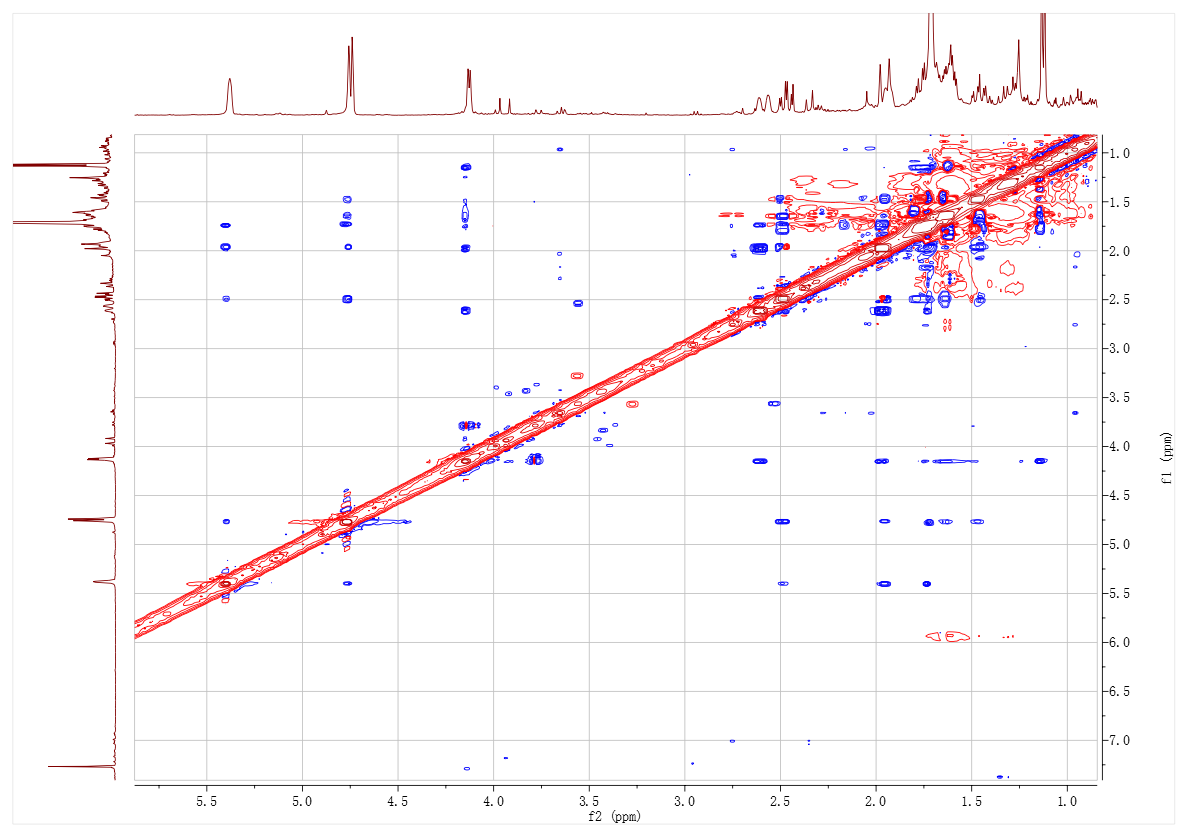


****Figure S21. NOESY spectrum of the new compound **6**


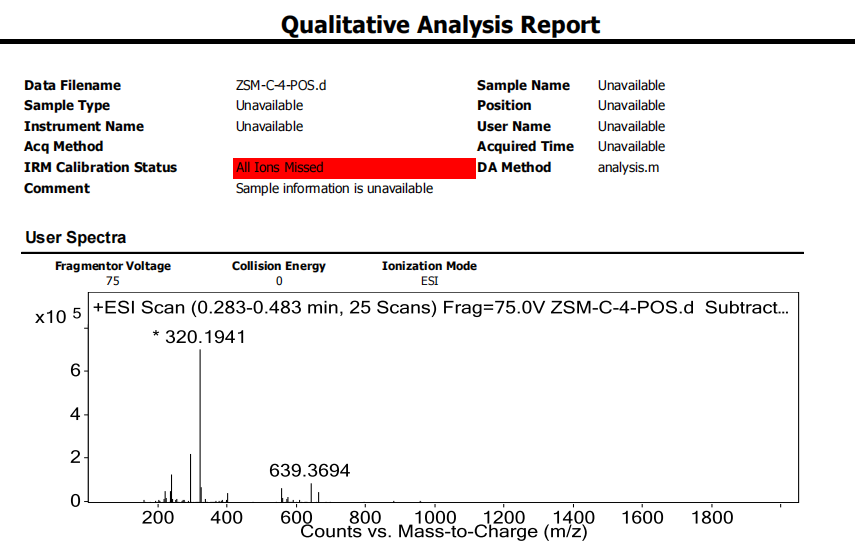


Figure S22. HRESI-MS spectrum of the new compound **7**


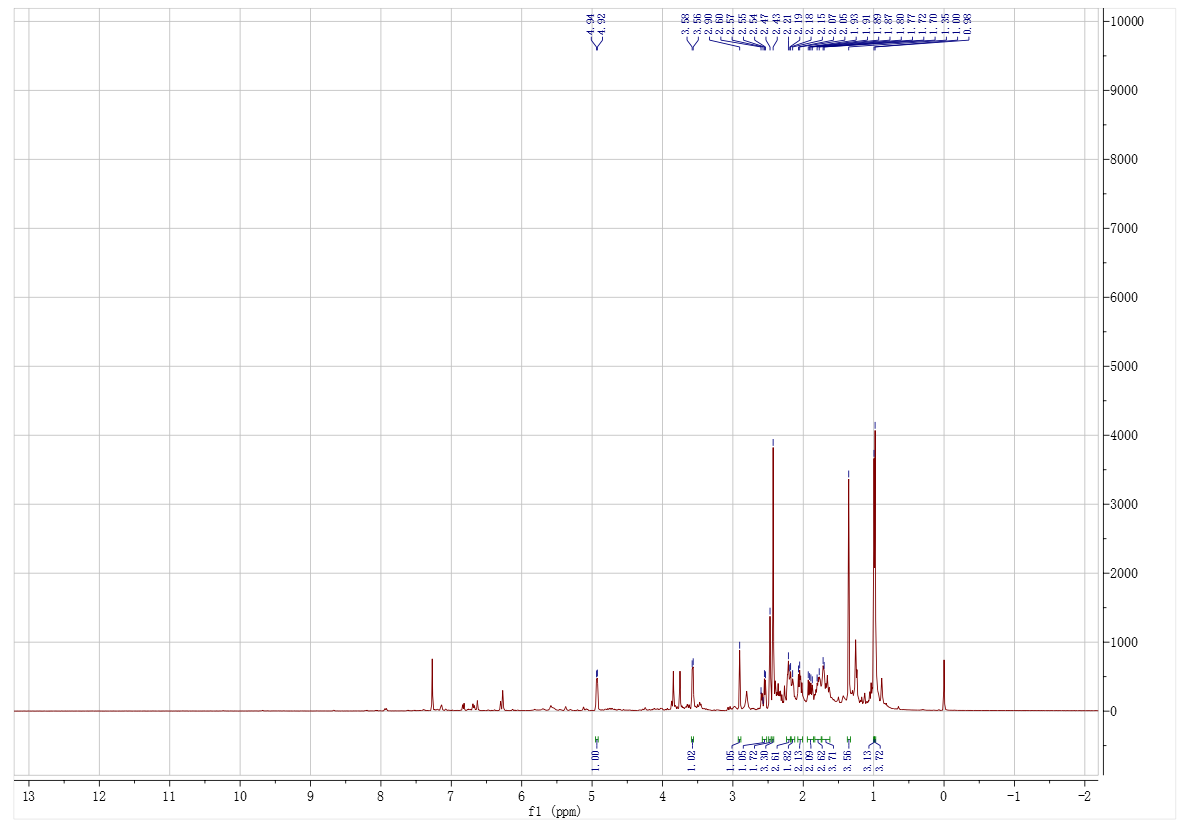


Figure S23. ^1^H NMR (600 MHz, CD_3_OD) spectrum of the new compound **7**


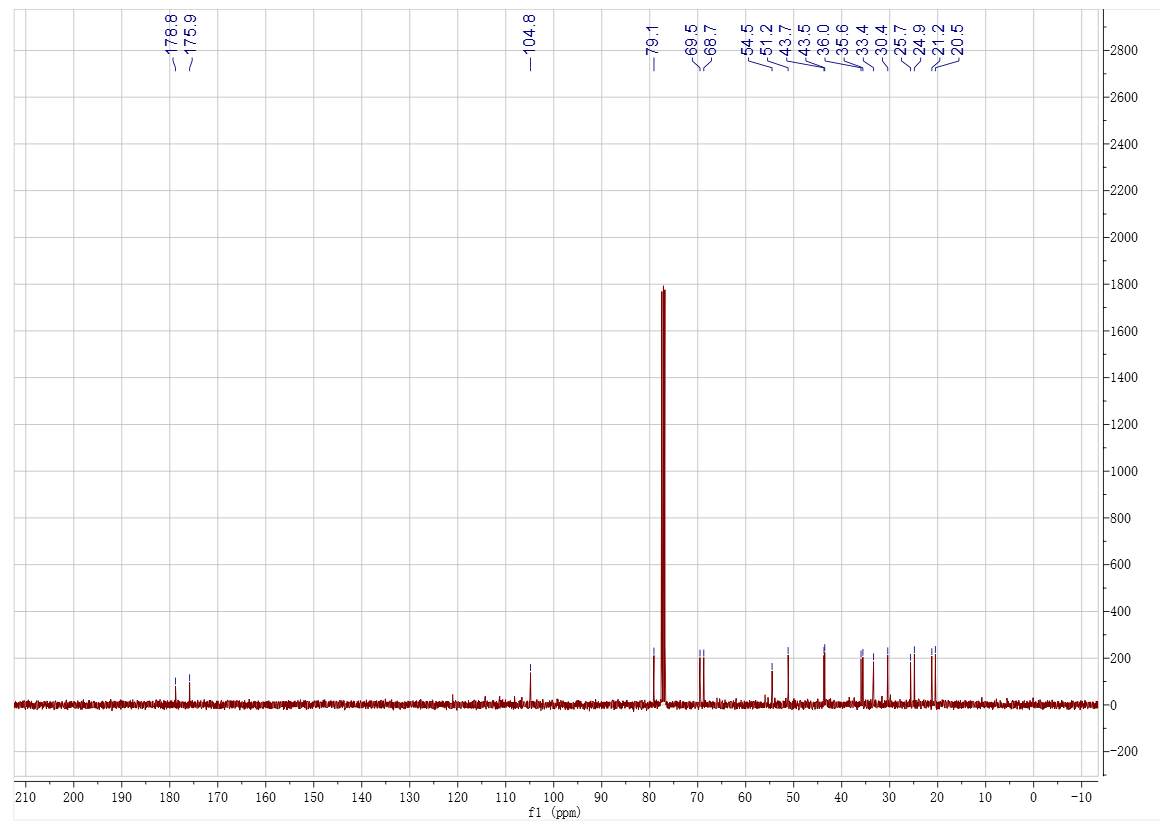


Figure S24. ^13^C NMR (150 MHz, CD_3_OD) spectrum of the new compound **7**


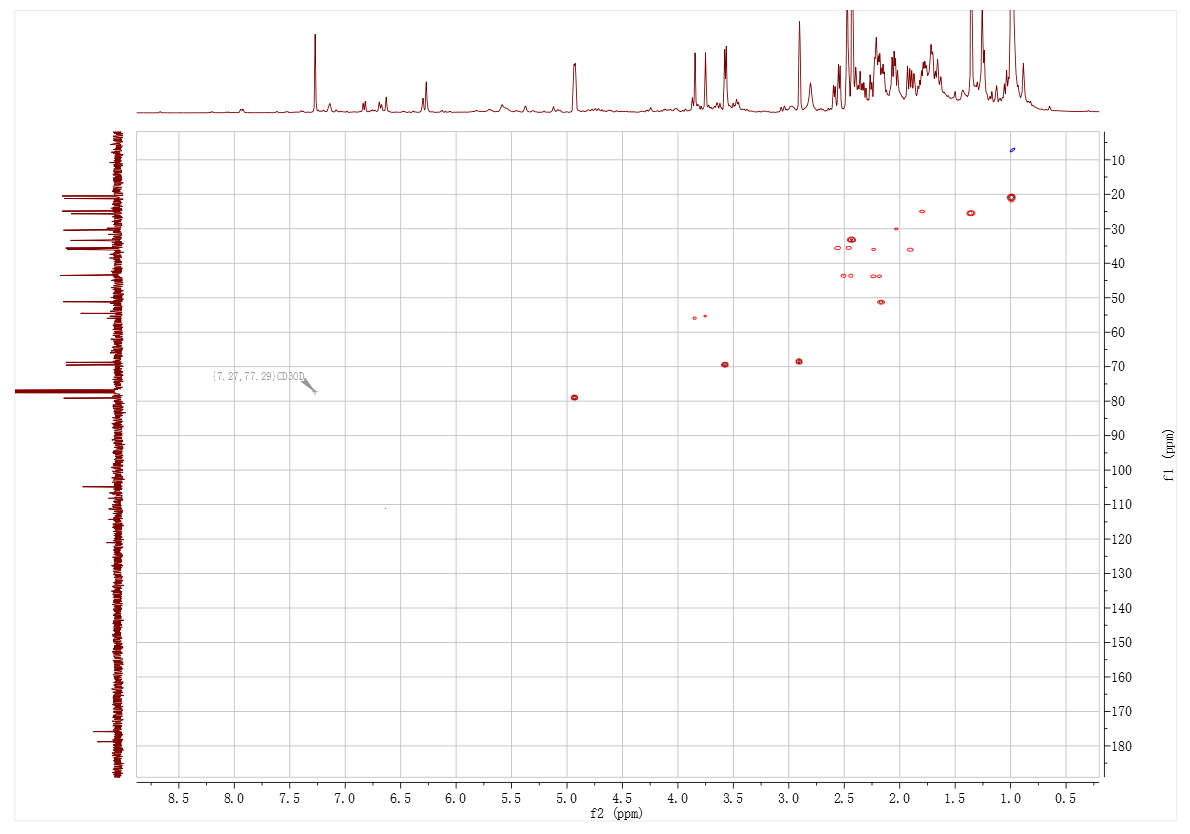


Figure S25. HSQC spectrum of the new compound **7**


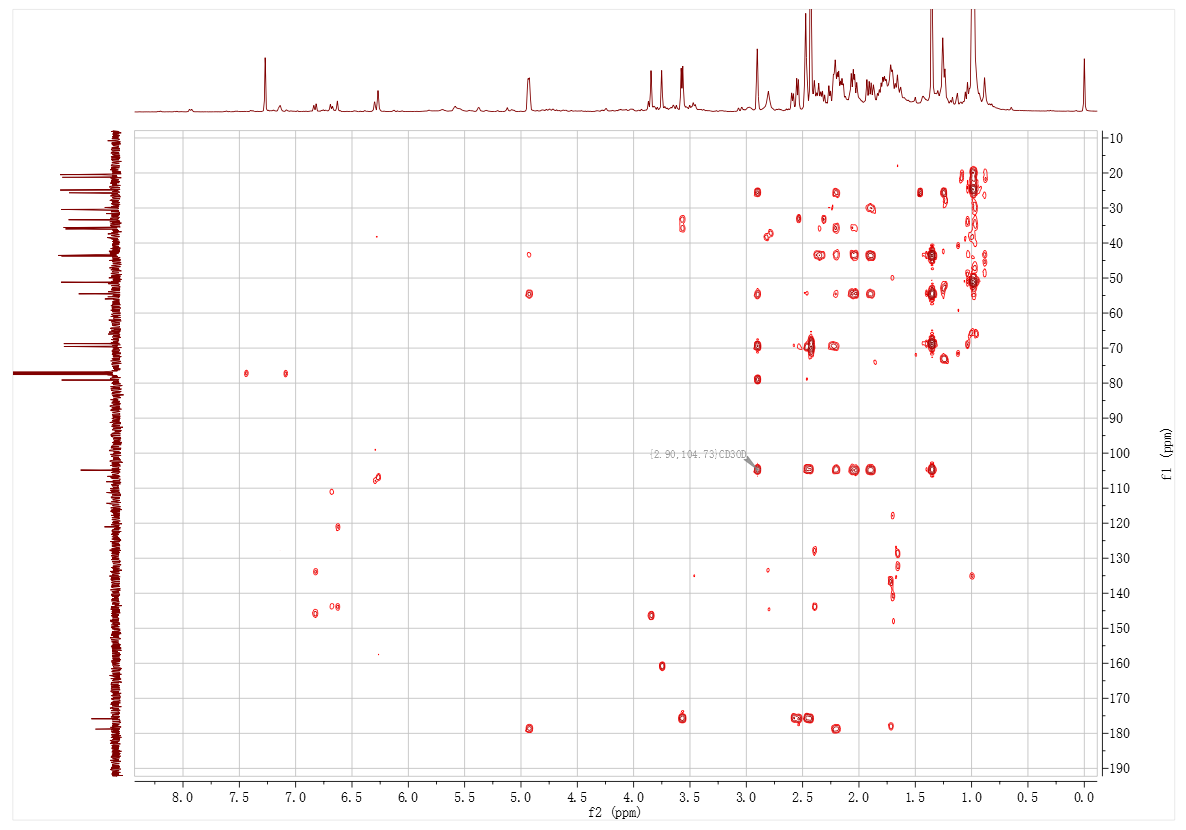


Figure S26. HMBC spectrum of the new compound **7**


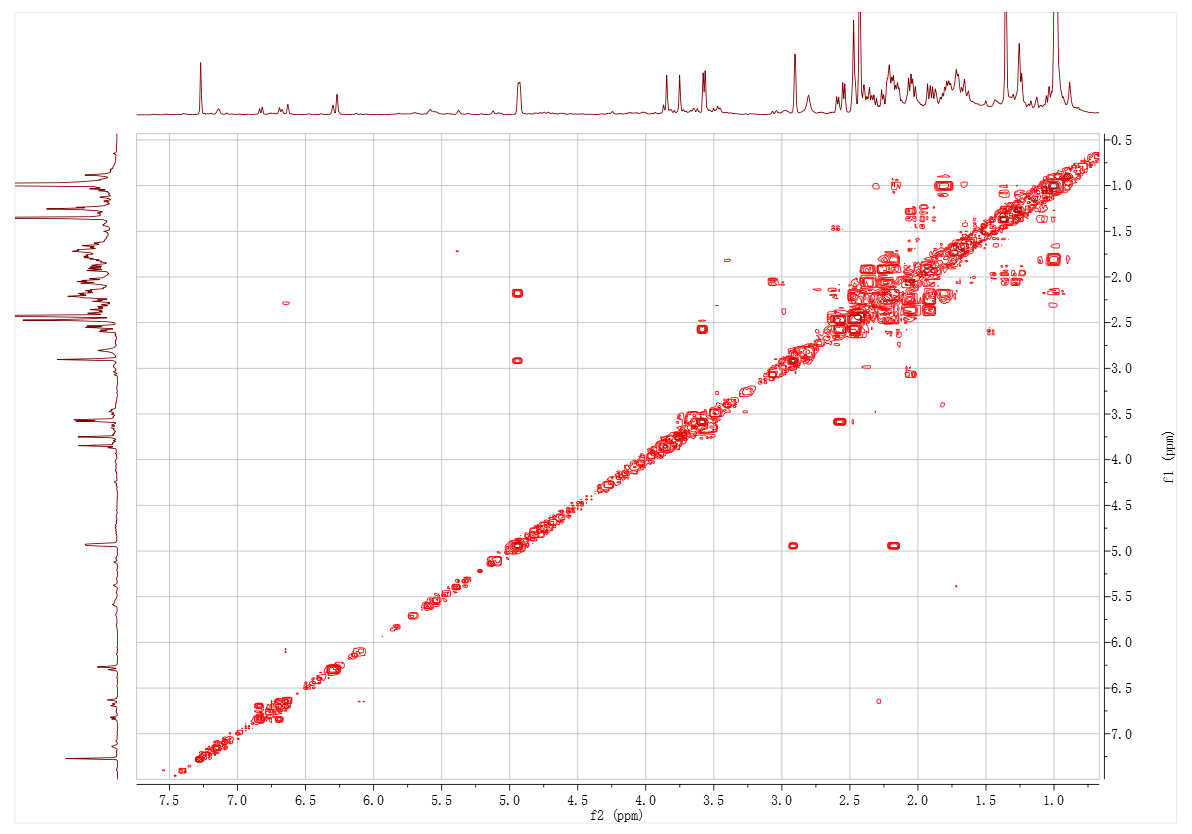


Figure S27. COSY spectrum of the new compound **7**


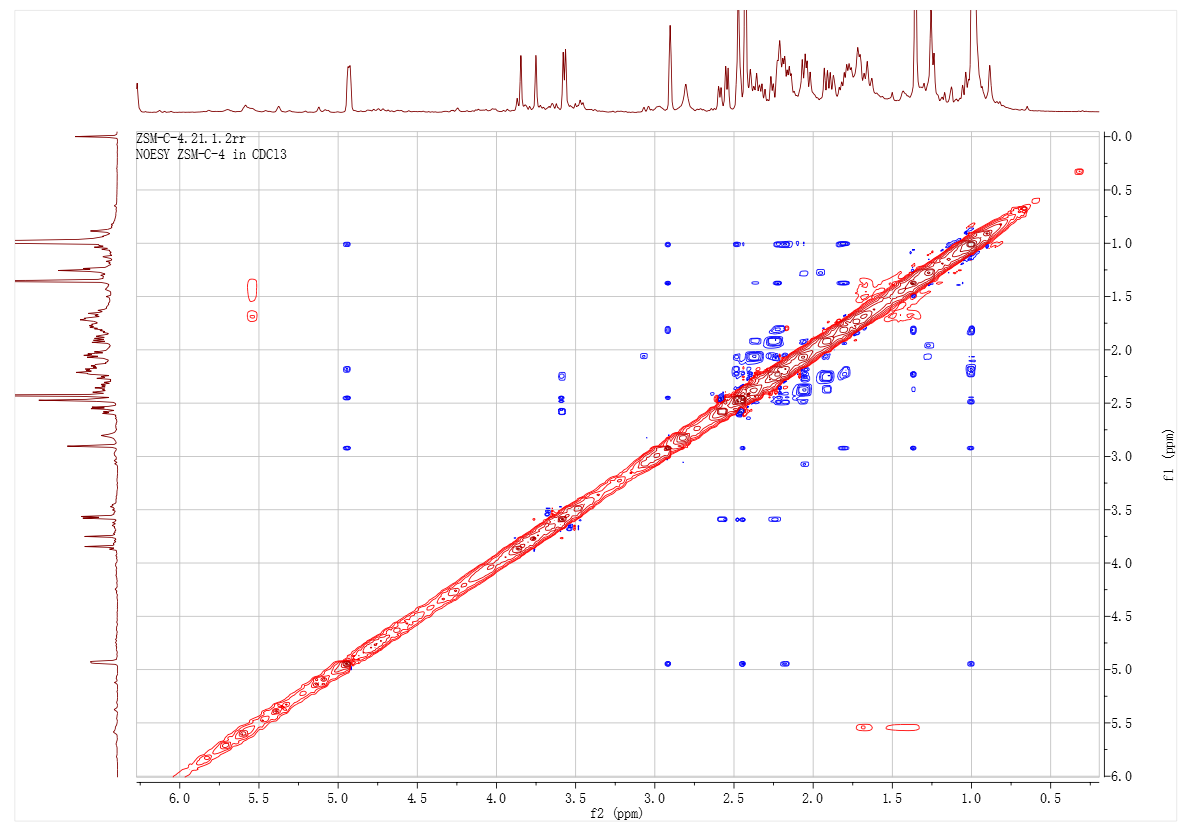


Figure S28. NOESY spectrum of the new compound **7**


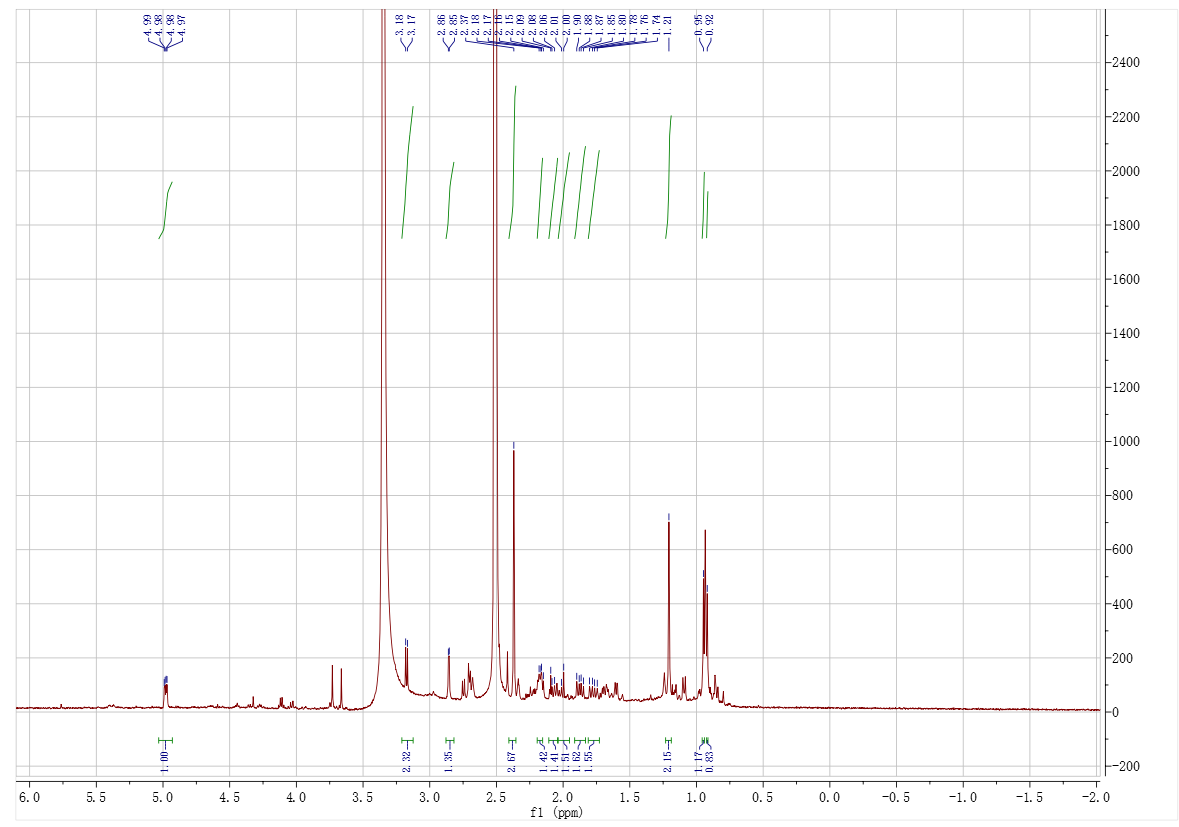


Figure S29. ^1^H NMR (DMSO-*d*_6_) spectrum of the new compound **7**


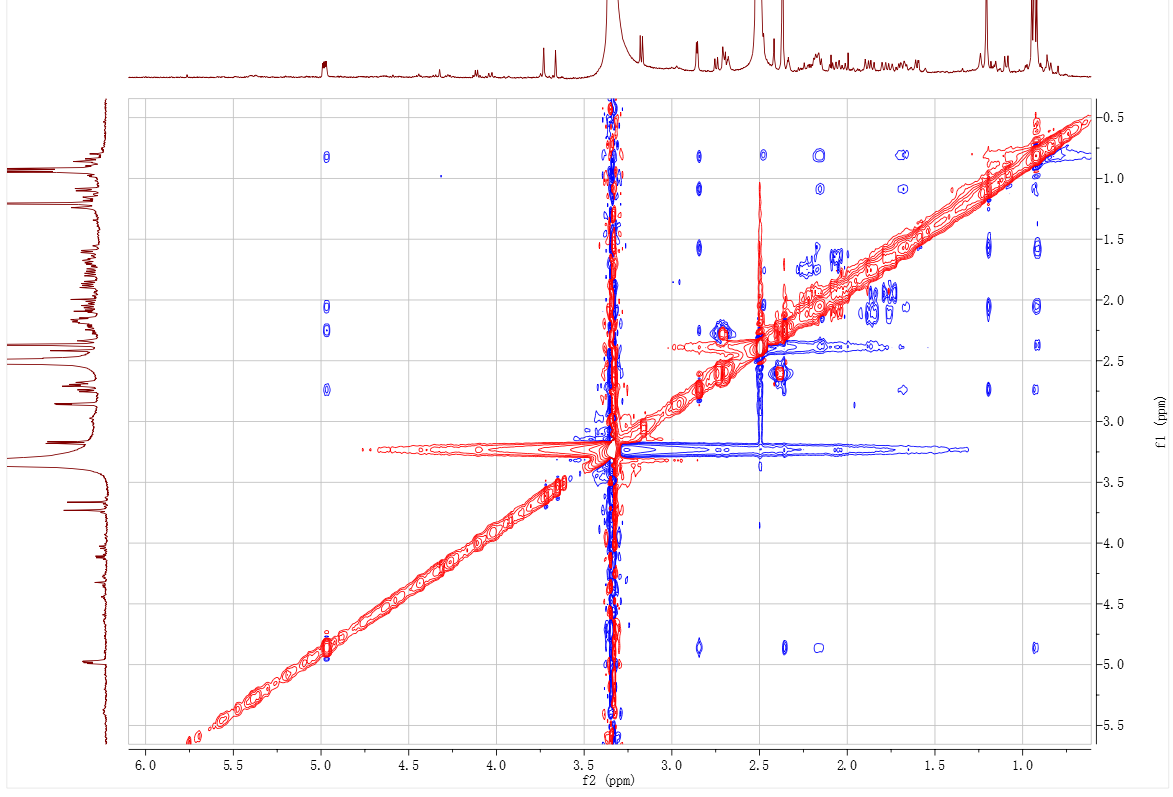


Figure S30. NOESY (DMSO-*d*_6_) spectrum of the new compound **7**


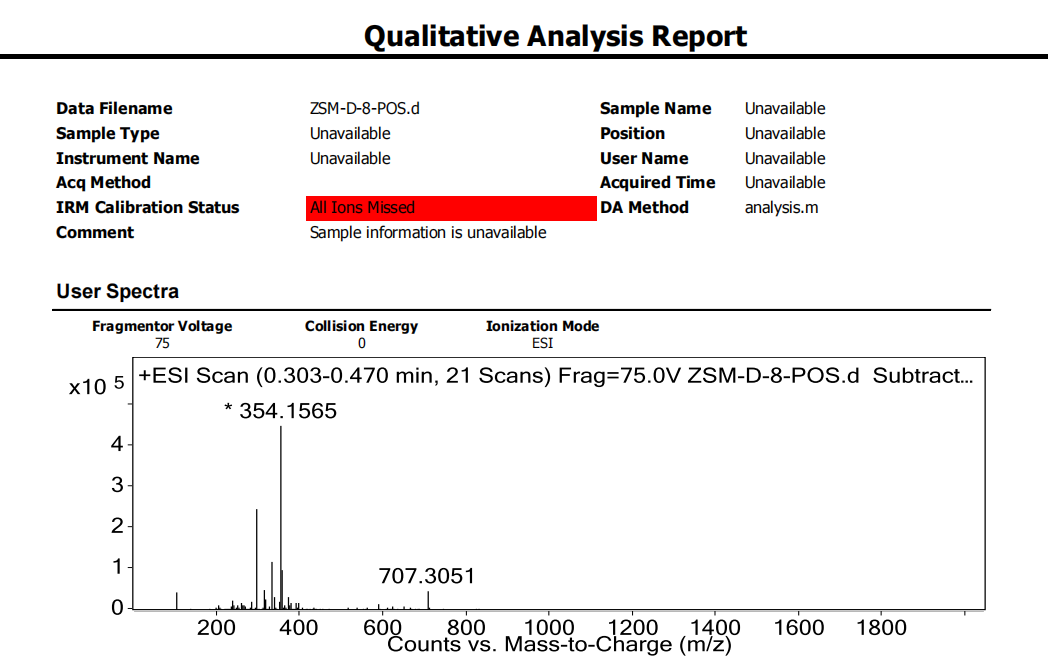


Figure S31. HRESI-MS spectrum of the new compound **8**


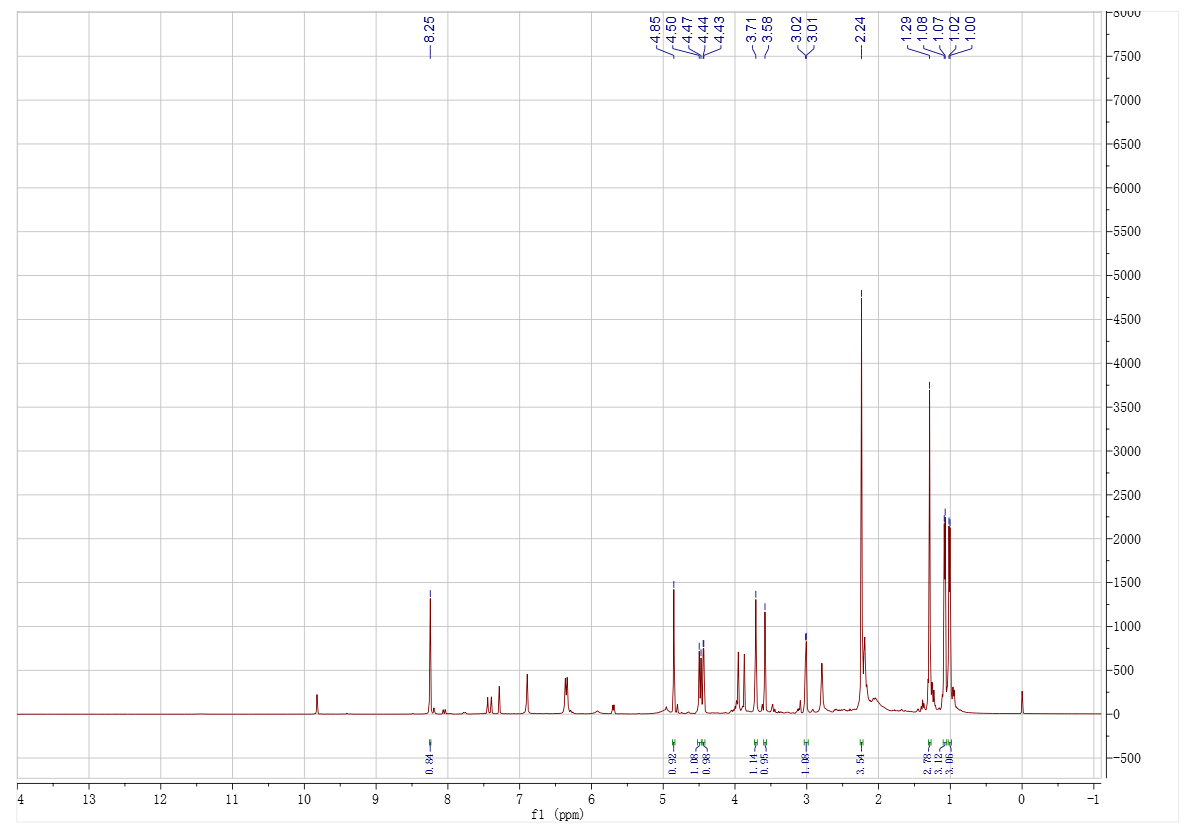


Figure S32. ^1^H NMR (600 MHz, CD_3_OD) spectrum of the new compound **8**


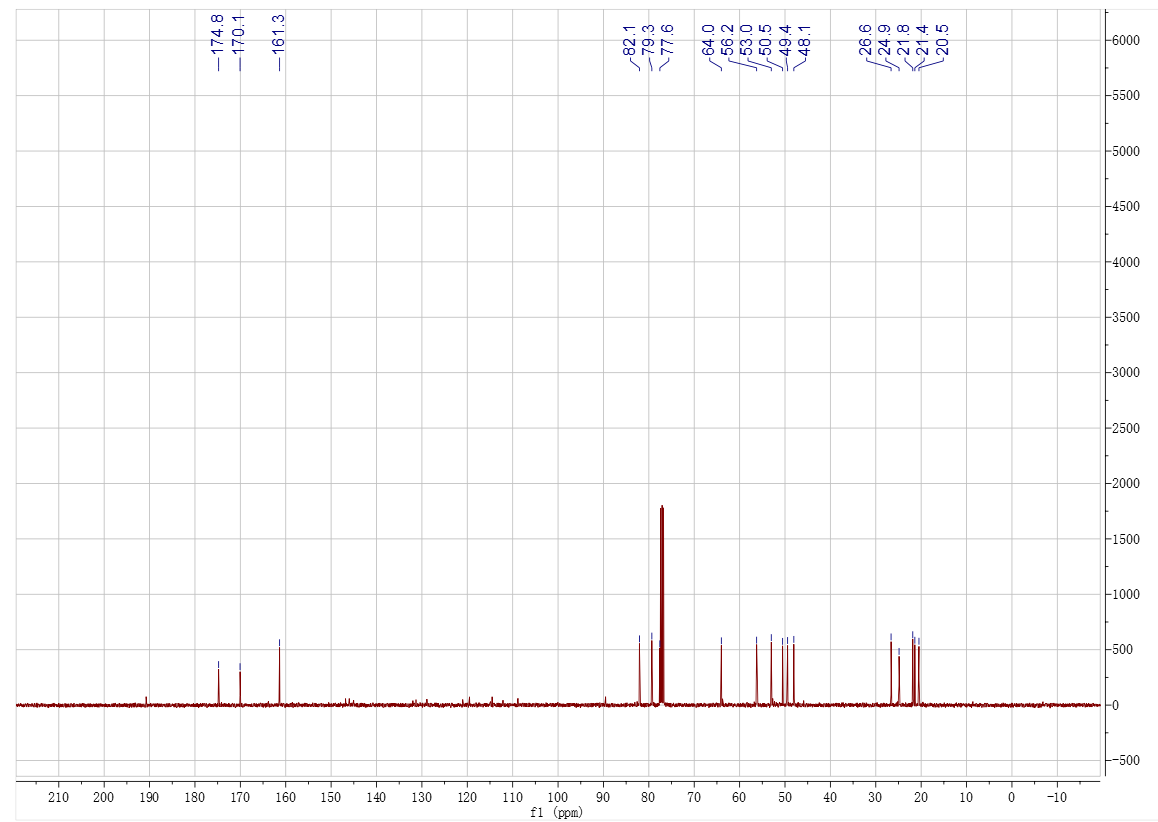


Figure S33. ^13^C NMR (150 MHz, CD_3_OD) spectrum of the new compound **8**


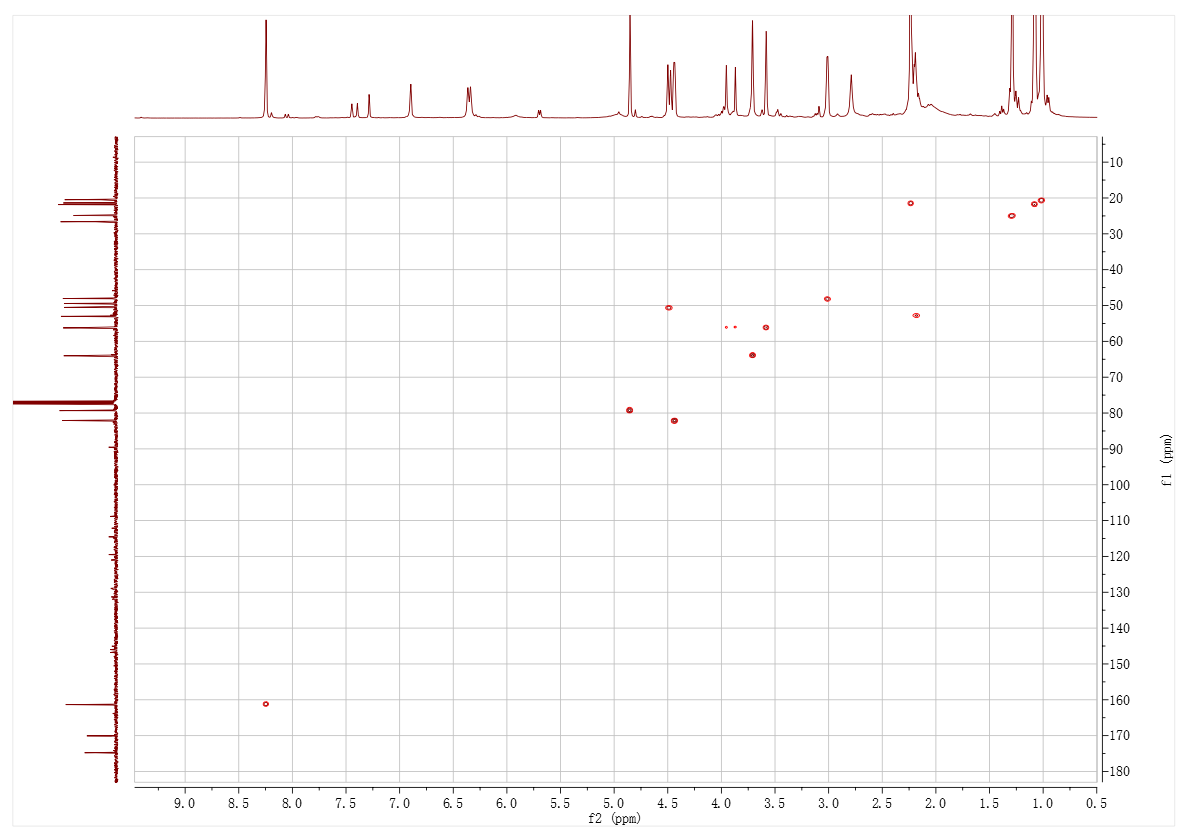


Figure S34. HSQC spectrum of the new compound **8**


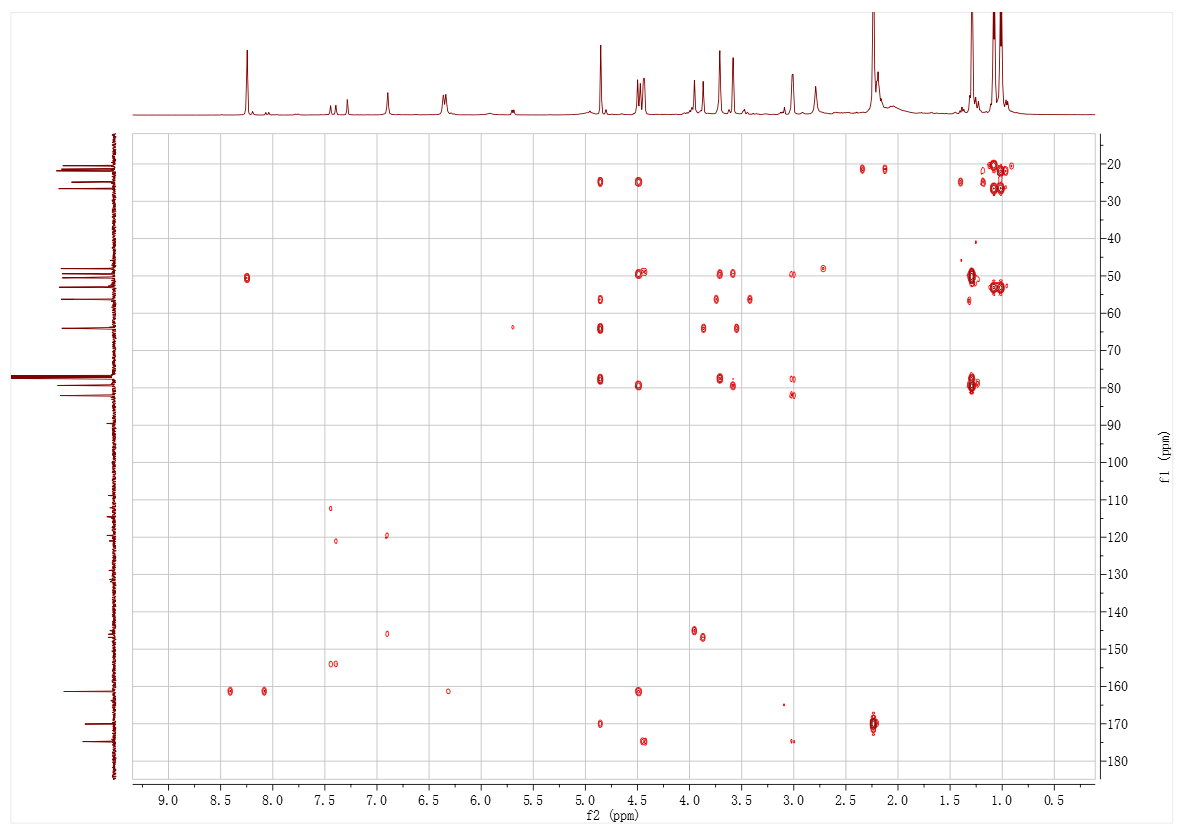


Figure S35. HMBC spectrum of the new compound **8**


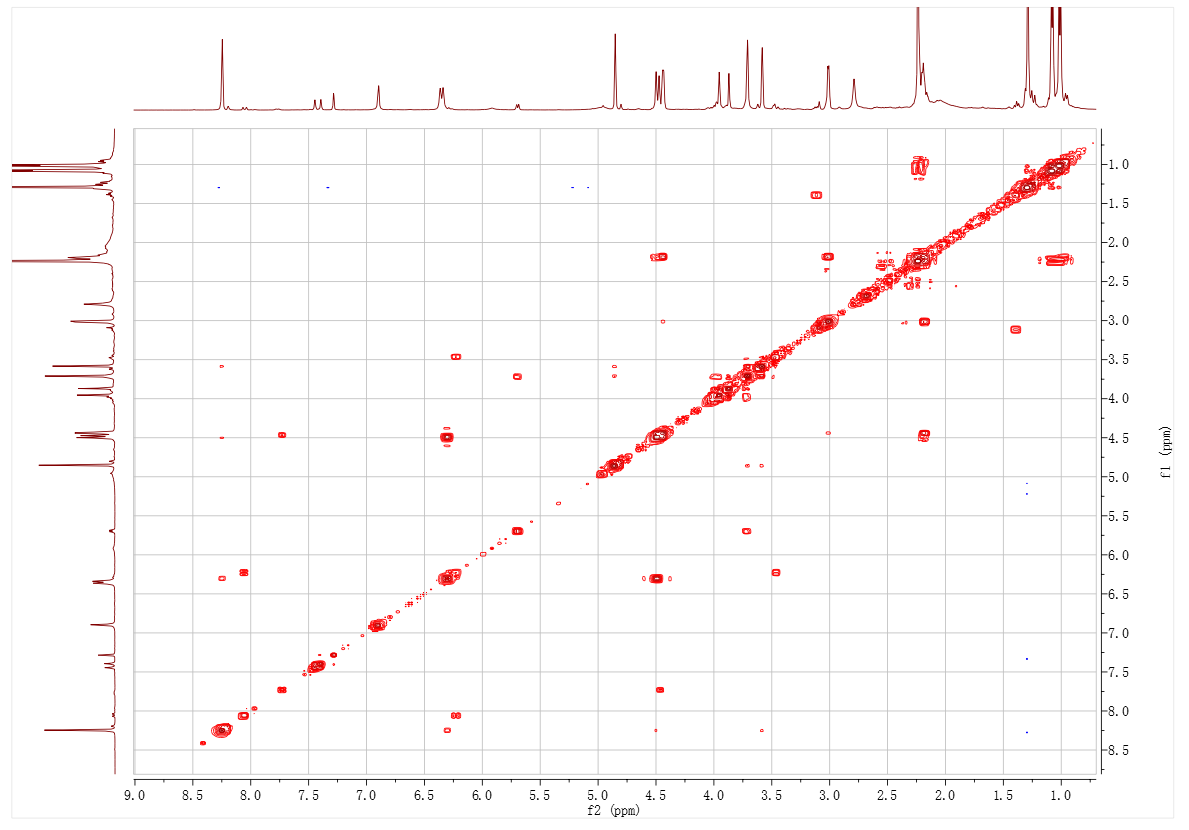


Figure S36. COSY spectrum of the new compound **8**


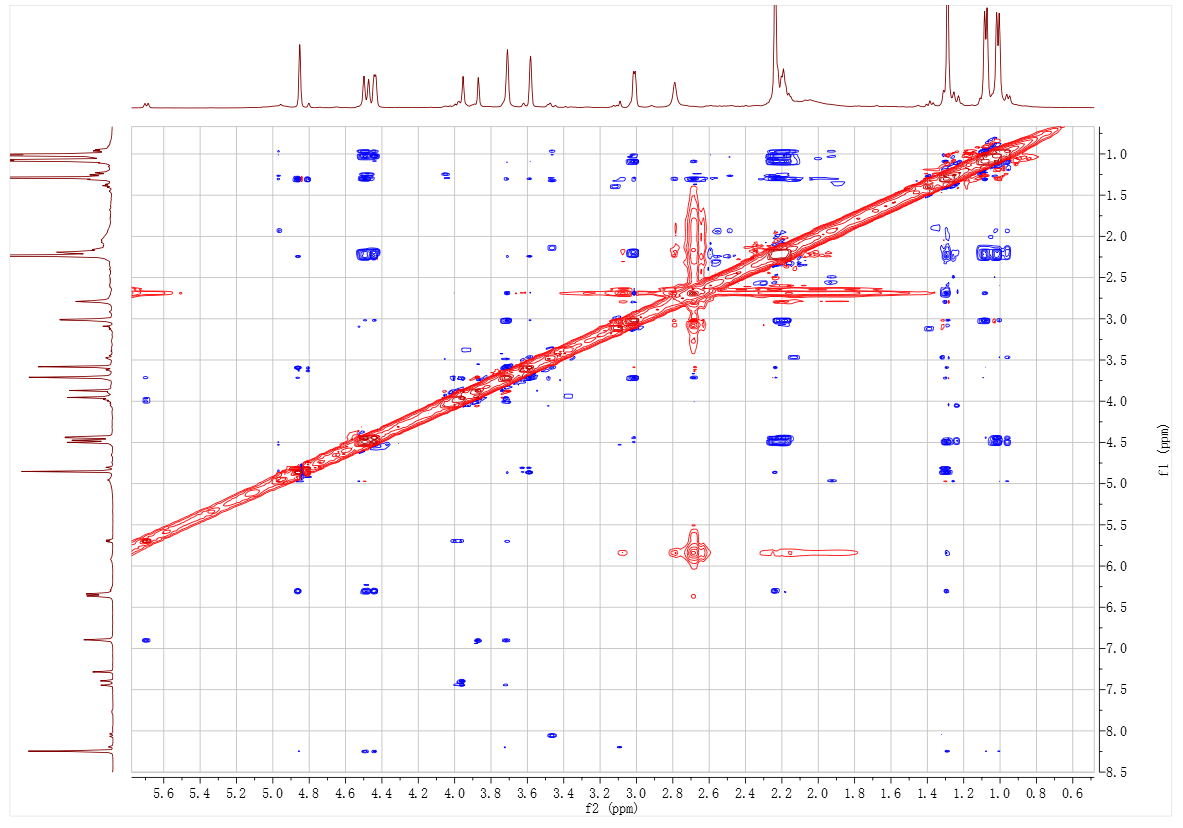


Figure S37. NOESY spectrum of the new compound **8.**


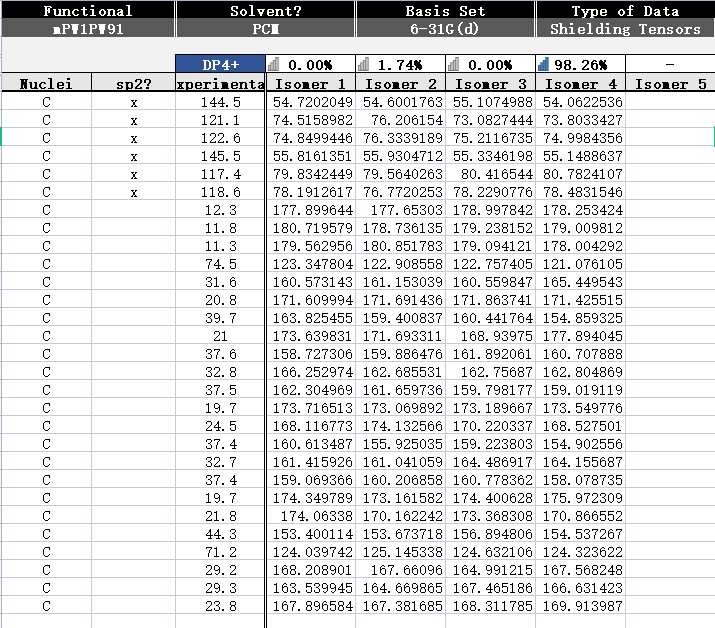


Figure S38. The data of DP4plus method of compound **1**


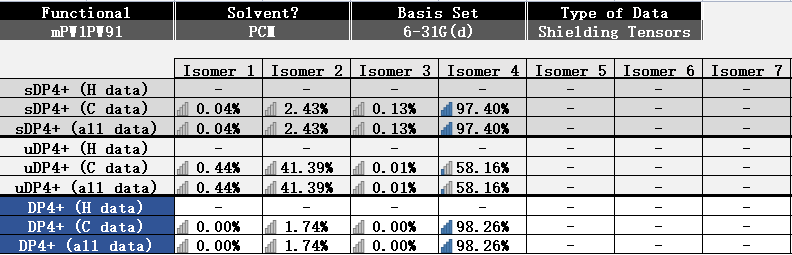


Figure S39. Comparison of the experimental ^13^C NMR data and mean absolute error of compound **1**


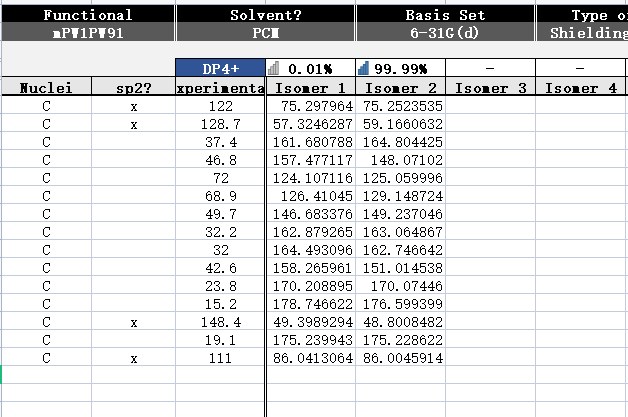


Figure S40. The data of DP4plus method of compound **1**


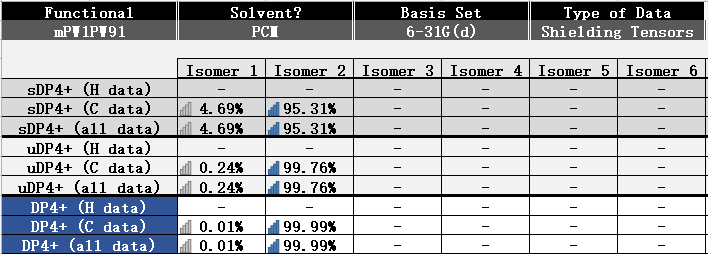


Figure S41. Comparison of the experimental ^13^C NMR data and mean absolute error of compound **6**

Figure S42. Chemical structure of the calculated configurations of compound **1** and **6**.

| Configuration | DP4+(%) | *R*^2^ | RMSE |
| --- | --- | --- | --- |
| **1a** | 0.00 | 0.9956 | 2.85 |
| **1b** | 1.74 | 0.9967 | 2.44 |
| **1c** | 0.00 | 0.9958 | 2.75 |
| **1d** | 98.26 | 0.9970 | 2.37 |
| **6a** | 0.01 | 0.9924 | 4.82 |
| **6b** | 99.99 | 0.9942 | 3.98 |

Figure S43. Statistics of Ordinary Least Squares (OLS) Linear Regression of experimental and computed ^13^C-NMR chemical shifts.

# Energies and Populations

# Energies at M062X/6-31G(d) theory level

Energies of the calculated configuration of **1a.**

**Table S1** Energies and populations of conformers of the calculated configuration.

| Conformer | Population（%） | E（Hartree） |
| --- | --- | --- |
| **1** | 14.09 | -1360.302843 |
| **2** | 6.55 | -1360.30212 |
| **5** | 4.94 | -1360.301854 |
| **6** | 1.52 | -1360.300741 |
| **7** | 5.56 | -1360.301967 |
| **10** | 3.53 | -1360.301539 |
| **11** | 3.27 | -1360.301466 |
| **12** | 2.79 | -1360.301317 |
| **17** | 3.51 | -1360.301534 |
| **18** | 1.62 | -1360.300806 |
| **19** | 1.23 | -1360.300547 |
| **21** | 2.15 | -1360.301072 |
| **22** | 3.27 | -1360.301466 |
| **23** | 4.25 | -1360.301714 |
| **25** | 4.1 | -1360.301679 |
| **27** | 2.5 | -1360.301213 |
| **32** | 13.15 | -1360.302778 |
| **45** | 1.48 | -1360.300719 |
| **49** | 2.79 | -1360.301316 |
| **68** | 17.68 | -1360.303058 |

Energies of the calculated configuration of **1b.**

**Table S2** Energies and populations of conformers of the calculated configuration.

| Conformer | Population（%） | E（Hartree） |
| --- | --- | --- |
| **1** | 54.53 | -1360.302444 |
| **2** | 34.85 | -1360.302021 |
| **7** | 2.62 | -1360.29958 |
| **9** | 3.13 | -1360.299749 |
| **11** | 1.19 | -1360.298832 |
| **15** | 1.16 | -1360.298814 |
| **25** | 1.14 | -1360.298798 |
| **27** | 1.37 | -1360.29897 |

Energies of the calculated configuration of **1c.**

**Table S3** Energies and populations of conformers of the calculated configuration.

| Conformer | Population（%） | E（Hartree） |
| --- | --- | --- |
| **1** | 2.52 | -1360.299991 |
| **2** | 2.61 | -1360.300023 |
| **4** | 3.62 | -1360.300332 |
| **9** | 1.28 | -1360.299351 |
| **10** | 86.23 | -1360.303323 |
| **11** | 2.61 | -1360.300022 |
| **21** | 1.13 | -1360.299235 |

Energies of the calculated configuration of **1d.**

**Table S4** Energies and populations of conformers of the calculated configuration.

| Conformer | Population（%） | E（Hartree） |
| --- | --- | --- |
| **1** | 44.98 | -1360.305371 |
| **3** | 2.52 | -1360.302654 |
| **5** | 3.34 | -1360.302919 |
| **8** | 2.33 | -1360.302577 |
| **9** | 4.29 | -1360.303154 |
| **15** | 36.29 | -1360.305169 |
| **23** | 2.96 | -1360.302804 |
| **29** | 3.29 | -1360.302904 |

Energies of the calculated configuration of **6a.**

**Table S5** Energies and populations of conformers of the calculated configuration.

| Conformer | Population（%） | E（Hartree） |
| --- | --- | --- |
| **1** | 10.85 | -736.141245 |
| **2** | 9.09 | -736.1410783 |
| **3** | 42.87 | -736.1425408 |
| **4** | 9.49 | -736.1411184 |
| **5** | 1.55 | -736.1394081 |
| **6** | 2.58 | -736.1398886 |
| **7** | 9.79 | -736.1411475 |
| **8** | 1.37 | -736.1392939 |
| **9** | 6.73 | -736.1407945 |
| **10** | 2.51 | -736.1398647 |
| **11** | 1.63 | -736.1394588 |
| **13** | 1.53 | -736.1393981 |

Energies of the calculated configuration of **6b.**

**Table S6** Energies and populations of conformers of the calculated configuration.

| Conformer | Population（%） | E（Hartree） |
| --- | --- | --- |
| **1** | 20.07 | -736.141764 |
| **2** | 23.91 | -736.141929 |
| **3** | 24.07 | -736.1419353 |
| **4** | 3.81 | -736.1401966 |
| **5** | 2.68 | -736.1398658 |
| **6** | 2.63 | -736.1398457 |
| **7** | 18.96 | -736.1417104 |
| **8** | 3.88 | -736.1402136 |
